# Insulin treatment and clinical outcomes in patients with diabetes and heart failure with preserved ejection fraction

**DOI:** 10.1002/ejhf.1535

**Published:** 2019-07-04

**Authors:** Li Shen, Rasmus Rørth, Deborah Cosmi, Søren Lund Kristensen, Mark C. Petrie, Franco Cosmi, Roberto Latini, Lars Køber, Inder S. Anand, Peter E. Carson, Christopher B. Granger, Michel Komajda, Robert S. McKelvie, Scott D. Solomon, Lidia Staszewsky, Karl Swedberg, Thao Huynh, Michael R. Zile, Pardeep S. Jhund, John J.V. McMurray

**Affiliations:** ^1^ Department of Cardiology, The Second Affiliated Hospital, College of Medicine Zhejiang University Hangzhou China; ^2^ BHF Cardiovascular Research Centre University of Glasgow Glasgow UK; ^3^ Rigshospitalet Copenhagen University Hospital Copenhagen Denmark; ^4^ Department of Cardiology Hospital of Gubbio Gubbio Italy; ^5^ Department of Cardiology Hospital of Cortona Cortona Italy; ^6^ Department of Cardiovascular Research IRCCS Istituto di Ricerche Farmacologiche Mario Negri Milan Italy; ^7^ Department of Medicine University of Minnesota Medical School and VA Medical Center Minneapolis MN USA; ^8^ Department of Cardiology Washington VA Medical Center Washington DC USA; ^9^ Duke Clinical Research Institute Duke University Durham NC USA; ^10^ Department of Cardiology Hospital Saint Joseph Paris France; ^11^ Western University London Ontario Canada; ^12^ Department of Medicine, Brigham and Women's Hospital Harvard Medical School Boston MA USA; ^13^ Department of Molecular and Clinical Medicine University of Gothenburg Gothenburg Sweden; ^14^ National Heart and Lung Institute Imperial College London London UK; ^15^ McGill Health University Center Division of Cardiology Montreal Quebec Canada; ^16^ Medical University of South Carolina and Ralph H. Johnston Veterans Administration Medical Center Charleston SC USA

**Keywords:** Heart failure, Diabetes mellitus, Insulin

## Abstract

**Aims:**

Insulin causes sodium retention and hypoglycaemia and its use is associated with worse outcomes in heart failure (HF) with reduced ejection fraction. We have investigated whether this is also the case in HF with preserved ejection fraction (HFpEF).

**Methods and results:**

We examined the association between diabetes/diabetes treatments and the risk of the primary composite of cardiovascular death or HF hospitalization, as well as other outcomes in adjusted analyses in CHARM‐Preserved (left ventricular ejection fraction ≥ 45%), I‐Preserve and TOPCAT (Americas) pooled. Of 8466 patients, 2653 (31%) had diabetes, including 979 (37%) receiving insulin. Patients receiving insulin were younger, had a higher body mass index, prevalence of ischaemic aetiology, N‐terminal pro‐B‐type natriuretic peptide and use of diuretics, worse New York Heart Association class and signs and symptoms, and worse quality of life and renal function, compared to patients with diabetes not on insulin. Among the 1398 patients with echocardiographic data, insulin use was associated with higher left ventricular end‐diastolic pressure and more diastolic dysfunction than in other participants. The primary outcome occurred at a rate of 6.3 per 100 patient‐years in patients without diabetes, and 10.2 and 17.1 per 100 patient‐years in diabetes patients without and with insulin use, respectively [fully adjusted hazard ratio (aHR) insulin‐treated diabetes vs. other diabetes: 1.41, 95% confidence interval (CI) 1.23–1.63, *P* < 0.001]. The adjusted HR is 1.67 (95% CI 1.20–2.32, *p* = 0.002) for sudden death (insulin‐treated diabetes vs. other diabetes).

**Conclusions:**

Insulin use is associated with poor outcomes in HFpEF. Although we cannot conclude a causal association, the safety of insulin and alternative glucose‐lowering treatments in HF needs to be evaluated in clinical trials.

## Introduction

Type 2 diabetes mellitus is common in patients with heart failure and preserved ejection fraction (HFpEF), with a reported prevalence of around 30–40%.^1^ It has been suggested that diabetes plays a key pathophysiological role in the development of HFpEF, and is an independent predictor of adverse outcomes in HFpEF.^1^, ^2^, ^3^, ^4^ The importance of better understanding the safety and possible benefits of anti‐diabetes agents in patients with established heart failure (HF) is underscored by recent cardiovascular (CV) outcome trials in diabetes mellitus. Thiazolidinediones and, possibly, certain dipeptidyl peptidase‐4 inhibitors increase the risk of HF hospitalization,^5^, ^6^ glucagon‐like peptide‐1 receptor agonists have shown a neutral effect,^7^, ^8^, ^9^, ^10^ and more recently, three sodium–glucose co‐transporter 2 (SGLT2) inhibitors, empagliflozin, canagliflozin and dapagliflozin, have shown a significant reduction in HF hospitalization.^7^, ^8^, ^9^, ^10^

Insulin, a traditional second‐line treatment for type 2 diabetes, leads to sodium and water retention, weight gain and hypoglycaemia, with resultant sympathetic nervous system activation.^11^, ^12^ Theoretically, insulin may be detrimental to patients with concomitant HF, but this has yet to be tested in a randomized clinical trial in patients with established HF. In a post‐hoc analysis of the Candesartan in Heart failure: Assessment of Reduction in Mortality and morbidity (CHARM) trial, insulin therapy was associated with higher risks of mortality and HF hospitalization in patients with HF and reduced ejection fraction (HFrEF) and similar findings have been reported from other HFrEF clinical trials.^13^, ^14^ Likewise, adverse outcomes associated with insulin treatment were observed in HF patients included in a large administrative registry from Italy.^14^ However, little is known about the characteristics of and clinical outcomes in HFpEF patients with diabetes taking insulin.^15^ HFpEF may be a more insulin‐resistant state than HFrEF and it has been suggested that insulin plays a role in the myocardial changes characterizing the HFpEF phenotype.^16^ Therefore, we examined the relationship between baseline insulin therapy and clinical outcomes in patients enrolled in the three largest randomized trials in HFpEF.

## Methods

### Study population

For these analyses, we combined patients enrolled in the CHARM‐Preserved trial,^17^ the Irbesartan in Heart Failure with Preserved Ejection Fraction (I‐Preserve) trial^18^ and the Treatment of Preserved Cardiac Function Heart Failure with an Aldosterone Antagonist (TOPCAT) trial.^19^ The design and main results of these trials have been published. Briefly, CHARM‐Preserved compared candesartan with placebo in 3023 HF patients in New York Heart Association (NYHA) functional class II–IV with a left ventricular ejection fraction (LVEF) > 40%. Patients in NYHA class II were required to have had a hospital admission for a cardiac reason within the past 6 months.^17^ In I‐Preserve, 4128 patients aged ≥ 60 years in NYHA class II–IV with a LVEF ≥ 45% were randomized to receive irbesartan or placebo. Patients in NYHA class II were eligible if they had a HF hospitalization within the previous 6 months.^18^ In TOPCAT, 3445 patients aged ≥ 50 years in NYHA class II–IV with a LVEF ≥ 45% were randomized to receive spironolactone or placebo; patients were eligible if they had been hospitalized for HF within the past 12 months, or had an elevated plasma natriuretic peptide level within 60 days before randomization [i.e. B‐type natriuretic peptide (BNP) ≥ 100 pg/mL or N‐terminal pro‐BNP (NT‐proBNP) ≥ 360 pg/mL].^19^

For the present analysis, patients with a LVEF < 45% in CHARM‐Preserved were excluded to ensure a consistent LVEF entry threshold across trials. Patients from Russia or Georgia in TOPCAT were also excluded because of substantially lower event rates in this region of enrolment compared to those in the Americas and there was uncertainty about whether they had HF.^20^ All trials were approved by the ethics committee in each study centre and all patients gave written informed consent.

### Diabetes status and insulin use at baseline

In each trial, investigators completed a case report form in which they were asked to state whether patients had diabetes and, if so, how it was treated (i.e. with insulin, oral therapy and diet control). The specific type of diabetes (type 1 or type 2) was not recorded in any of the trials. The age of onset of diabetes was recorded in two of the three trials (i.e. CHARM‐Preserved and TOPCAT); of these, 66 (4.4%) patients were diagnosed under 20 years of age. We aimed to examine the effect of insulin use in patients with type 2 diabetes and HFpEF, and analyses excluding these 66 patients, presumably with type 1 diabetes, are presented in the online supplementary *Table* S1–S5 and *Figure* S1). Type 1 diabetes is clearly different because there is complete absence of pancreatic insulin production and exogenous insulin therapy is mandatory from the outset, unlike in type 2 diabetes.

### Outcomes

The primary outcome was a composite of CV death or HF hospitalization in CHARM‐Preserved, all‐cause death or protocol‐specified CV hospitalization in I‐Preserve, and a composite of CV death, HF hospitalization or aborted cardiac arrest in TOPCAT. For this analysis, we specified the following outcomes of interest: the composite of CV death or first HF hospitalization and its individual components, as well as all‐cause death. We also examined the two main modes of CV death, i.e. sudden death and pump failure death. In each trial, potential endpoints were adjudicated by an independent committee using similar pre‐specified criteria (the same committee adjudicated the events in two of the three trials, i.e. CHARM‐Preserved and TOPCAT).

### Statistical analyses

Baseline characteristics were summarized as means with standard deviations for continuous variables and frequencies with percentages for categorical variables. Differences in baseline characteristics according to diabetes status and insulin use were examined using ANOVA for continuous variables with Bonferroni correction for multiple comparisons and the χ^2^ test for categorical variables. Duration of diabetes, the Minnesota Living with Heart Failure (MLWHF) score, Kansas City Cardiomyopathy Questionnaire (KCCQ) clinical summary score and NT‐proBNP were not normally distributed and therefore were summarized as medians with interquartile ranges and analysed using the Kruskal–Wallis test with Dunn's test and a Bonferroni correction for multiple comparisons. Event rates for the outcomes of interest were calculated per 100 patient‐years of follow‐up and illustrated using Kaplan–Meier curves; cumulative incidence curves are presented in online supplementary *Figure* S1.

Cox proportional hazards regression analysis was used to calculate the hazard ratio (HR) for each outcome with the comparisons of insulin‐treated diabetes vs. no diabetes and non‐insulin‐treated diabetes vs. no diabetes. The proportional hazards regression analyses were also performed with adjustment for a number of confounding variables, including age, sex, race, heart rate, diastolic blood pressure, LVEF, NYHA class III/IV (vs. I/II), body mass index (BMI), HF hospitalization within the past 6 months, history of myocardial infarction, hypertension, or atrial fibrillation, estimated glomerular filtration rate (eGFR), and log‐transformed NT‐proBNP with simple imputation of eGFR and NT‐proBNP using the missing indicator method; sensitivity analyses including only patients with complete NT‐proBNP data are shown in online supplementary *Tables* S4 and S5). Within‐trial clustering was taken into consideration with the use of shared frailty models. The proportional‐hazards assumption was examined with the use of the Schoenfeld residuals.

As patients with diabetes treated with insulin tend to have a longer standing diabetes than those not on insulin, we compared the risk of each outcome in patients with diabetes on insulin with those not on insulin further adjusting for the duration of diabetes in addition to the confounders mentioned above.

For patients with available echocardiographic data (745 patients in I‐Preserve and 653 patients in TOPCAT, respectively), we compared the measurements of left ventricular structure and left ventricular systolic and diastolic properties according to diabetes status and insulin use at baseline.

A two‐sided *P*‐value < 0.05 was considered significant. All analyses were performed using Stata version 15 (Stata Corp., College Station, TX, USA).

## Results

Overall, 10 596 patients were enrolled in CHARM‐Preserved, I‐Preserve and TOPCAT. Of these, 450 patients from CHARM‐Preserved had a LVEF < 45% and therefore were excluded. A further 1678 patients enrolled from Russia/Georgia in TOPCAT and two patients with missing information on diabetes status were also excluded, leaving 8466 patients for analysis. A total of 2653 (31%) patients had diabetes at baseline, of which 979 (37%) patients were treated with insulin. Patients treated with insulin, on average, had a much longer history of diabetes (16 years) than those not on insulin (7 years) (*Table* 1).

**Table 1:** Patient characteristics according to baseline diabetes mellitus status and insulin use in the combined data sets of CHARM‐Preserved (left ventricular ejection fraction ≥ 45%), I‐Preserve and TOPCAT (Americas)

	No DM (*n* = 5813)	DM not on insulin (*n* = 1674)	DM on insulin (*n* = 979)	*P*‐value			
				Overall	DM not on insulin vs. no DM	DM on insulin vs. no DM	DM on insulin vs. not on insulin
Age, years	70.6 ± 9.4	69.9 ± 8.7	67.8 ± 8.4	<0.0001	0.017	<0.001	<0.001
Age at diabetes onset, years	–	57.8 ± 14.9	48.2 ± 14.8				<0.0001
Duration of diabetes, years	–	7 [3–13]	16 [10–24]				<0.0001
Male sex	2724 (46.9)	837 (50.0)	447 (45.7)	0.0408	0.023	0.486	0.031
Race				<0.0001	<0.001	<0.001	<0.001
White	5357 (92.2)	1429 (85.4)	808 (82.5)				
Black	248 (4.3)	126 (7.5)	118 (12.1)				
Asian	62 (1.1)	36 (2.2)	20 (2.0)				
Other	146 (2.5)	83 (5.0)	33 (3.4)				
Body mass index, kg/m^2^	29.4 ± 5.8	31.9 ± 6.7	34.0 ± 7.6	<0.0001	<0.001	<0.001	<0.001
Blood pressure, mmHg							
Systolic	134.6 ± 16.7	135.0 ± 16.6	134.0 ± 17.4	0.313	0.974	0.99	0.394
Diastolic	77.7 ± 10.3	76.4 ± 10.6	72.9 ± 11.1	<0.0001	<0.001	<0.001	<0.001
Heart rate, b.p.m.	70.5 ± 11.4	71.6 ± 11.0	72.0 ± 11.7	<0.0001	0.001	0.001	0.99
NYHA class III–IV	3262 (56.1)	998 (59.6)	600 (61.3)	0.0013	0.011	0.003	0.397
LVEF, %	58.2 ± 8.9	58.1 ± 8.8	58.1 ± 8.9	0.916	0.99	0.99	0.99
Aetiology, *n* (%)				<0.0001	0.081	<0.001	<0.001
Ischaemic	1688 (34.9)	467 (36.9)	268 (44.7)				
Hypertensive	2393 (49.5)	631 (49.9)	231 (38.5)				
Other	755 (15.6)	167 (13.2)	101 (16.8)				
HF duration				0.0017	0.001	0.109	0.374
≤ 1 year	2280 (47.2)	557 (44.1)	285 (47.5)				
> 1 and ≤ 5 years	1800 (37.3)	454 (35.9)	204 (34.0)				
> 5 years	752 (15.6)	253 (20.0)	111 (18.5)				
Medical history							
Current smoking	321 (11.4)	79 (9.0)	47 (7.3)	0.0036	0.047	0.003	0.249
HF hospitalization within the past 6 months	2309 (39.7)	723 (43.2)	476 (48.6)	<0.0001	0.011	<0.001	0.007
Myocardial infarction	1550 (26.7)	529 (31.6)	305 (31.2)	<0.0001	<0.001	0.004	0.811
Angina	2565 (44.1)	765 (45.7)	454 (46.4)	0.278	0.254	0.19	0.736
CABG or PCI	1118 (19.2)	449 (26.8)	377 (38.5)	<0.0001	<0.001	<0.001	<0.001
Hypertension	4566 (78.5)	1492 (89.1)	872 (89.1)	<0.0001	<0.001	<0.001	0.964
Atrial fibrillation	1914 (32.9)	528 (31.5)	279 (28.5)	0.019	0.287	0.006	0.1
Stroke	481 (8.3)	184 (11.0)	114 (11.6)	<0.0001	0.001	0.001	0.607
Dyslipidaemia	1773 (44.6)	748 (62.1)	539 (75.1)	<0.0001	<0.001	<0.001	<0.001
Medications							
Diuretics	4609 (79.4)	1433 (85.7)	873 (89.3)	<0.0001	<0.001	<0.001	0.008
Loop	3243 (55.8)	1102 (65.9)	779 (79.7)	<0.0001	<0.001	<0.001	<0.001
Thiazide	1708 (29.4)	434 (25.9)	217 (22.2)	<0.0001	0.006	<0.001	0.03
Calcium channel blocker	2080 (35.8)	668 (39.9)	408 (41.7)	<0.0001	0.002	<0.001	0.365
ACEI or ARB	3555 (61.2)	1187 (70.9)	743 (75.9)	<0.0001	<0.001	<0.001	0.005
MRA	1151 (19.8)	382 (22.8)	289 (29.5)	<0.0001	0.007	<0.001	<0.001
Beta‐blocker	3524 (60.7)	1036 (61.9)	671 (68.6)	<0.0001	0.36	<0.001	0.001
Digoxin	995 (17.1)	302 (18.1)	155 (15.8)	0.347	0.383	0.321	0.147
Antiarrhythmic agent	574 (9.9)	132 (7.9)	56 (5.7)	<0.0001	0.014	<0.001	0.036
Antiplatelet	3346 (57.6)	1060 (63.4)	652 (66.7)	<0.0001	<0.001	<0.001	0.086
Oral anticoagulant	1440 (24.8)	391 (23.4)	224 (22.9)	0.272	0.232	0.203	0.783
Lipid‐lowering agent	2062 (35.5)	833 (49.8)	634 (64.8)	<0.0001	<0.001	<0.001	<0.001
ECG findings							
Atrial fibrillation/flutter	1113 (19.2)	309 (18.5)	146 (15.0)	0.0079	0.495	0.002	0.023
QRS duration, milliseconds	90 [80–108]	94 [80–110]	94 [82–112]	0.0006	0.0357	0.0007	0.2218
Bundle branch block	857 (14.8)	276 (16.5)	167 (17.2)	0.063	0.087	0.055	0.65
Left bundle branch block	247 (8.2)	59 (7.4)	30 (8.8)	0.662	0.446	0.705	0.413
Right bundle branch block	185 (6.2)	70 (8.8)	28 (8.3)	0.019	0.009	0.138	0.765
Left ventricular hypertrophy	1311 (22.6)	344 (20.6)	148 (15.2)	<0.0001	0.073	<0.001	0.001
Symptoms and signs							
Dyspnoea on exertion	2754 (97.7)	857 (97.5)	622 (97.2)	0.741	0.736	0.45	0.71
Orthopnoea	572 (20.4)	244 (27.9)	245 (38.5)	<0.0001	<0.001	<0.001	<0.001
Paroxysmal nocturnal dyspnoea	320 (11.4)	124 (14.3)	117 (18.5)	<0.0001	0.023	<0.001	0.028
Dyspnoea at rest	142 (7.7)	58 (12.3)	44 (16.9)	<0.0001	0.001	<0.001	0.091
Jugular venous distention	533 (9.3)	181 (11.0)	123 (12.9)	0.0009	0.037	<0.001	0.146
Oedema	2759 (47.5)	905 (54.1)	626 (63.9)	<0.0001	<0.001	<0.001	<0.001
Rales	1258 (21.7)	370 (22.2)	235 (24.2)	0.214	0.646	0.08	0.241
Third heart sound	299 (6.2)	99 (7.8)	53 (8.8)	0.012	0.036	0.013	0.458
Hepatomegaly	647 (13.4)	207 (16.4)	63 (10.6)	0.002	0.006	0.054	0.001
Health‐related quality of life							
Minnesota Living With Heart Failure score	40 [26–56]	45 [27–62]	51 [33–70]	<0.0001	0.0001	<0.0001	0.0004
KCCQ clinical summary score	65 [46–80]	58 [42–76]	52 [33–68]	<0.0001	0.0006	<0.0001	0.0002
Laboratory measurements							
eGFR, mL/min/1.73 m^2^	71.5 ± 22.2	71.4 ± 24.8	62.7 ± 23.9	<0.0001	0.99	<0.001	<0.001
eGFR < 60 mL/min/1.73 m^2^	1451 (32.4)	518 (37.0)	457 (53.3)	<0.0001	0.002	<0.001	<0.001
Haemoglobin, g/dL	13.9 ± 1.6	13.5 ± 1.7	12.9 ± 1.7	<0.0001	<0.001	<0.001	<0.001
NT‐proBNP, pg/mL	364 [139–1017]	430 [167–1041]	581 [207–1336]	<0.0001	0.174	<0.0001	0.0024

Values are given as mean ± standard deviation, *n* (%), or median [interquartile range].

ACEI, angiotensin‐converting enzyme inhibitor; ARB, angiotensin receptor blocker; CABG, coronary artery bypass grafting; DM, diabetes mellitus; ECG, electrocardiogram; eGFR, estimated glomerular filtration rate; HF, heart failure; KCCQ, Kansas City Cardiomyopathy Questionnaire; LVEF, left ventricular ejection fraction; MRA, mineralocorticoid receptor antagonist; NT‐proBNP, N‐terminal pro‐B‐type natriuretic peptide; NYHA, New York Heart Association; PCI, percutaneous coronary intervention.

Age at diabetes onset and duration of diabetes were available in 716 (98%) patients from CHARM‐Preserved and 779 (99%) from TOPCAT; NT‐proBNP was available in 3838 (45%) patients; eGFR was available in 6735 (80%) patients; haemoglobin was available in 5735 (68%) patients. Current smoking, dyspnoea on exertion, orthopnoea, paroxysmal nocturnal dyspnoea and dyspnoea at rest were not recorded in I‐Preserve. Dyslipidaemia was not recorded in CHARM‐Preserved. Third heart sound and hepatomegaly were not recorded in TOPCAT. Left bundle branch block and right bundle branch block were only available in I‐Preserve.

Minnesota Living With Heart Failure score was available in 783 (30%) patients from CHARM‐Preserved and 3148 (76%) patients from I‐Preserve, and possible scores range from 0 to 105, with lower scores indicating a better quality of life.

KCCQ clinical summary score was available in 1726 (98%) patients from TOPCAT, and possible scores range from 0 to 100, with higher scores indicating better health‐related quality of life.

### Baseline characteristics

*Table* 1 shows the patient characteristics according to diabetes status and insulin use at baseline. Patients with diabetes who received insulin were younger (68 years) and less often of white race (83%), compared to patients with diabetes not on insulin (70 years and 85%, respectively) or patients without diabetes (71 years and 92%, respectively). The average BMI was highest in insulin‐treated patients with diabetes (34 kg/m^2^), intermediate in non‐insulin‐treated patients with diabetes (32 kg/m^2^), and lowest in those without diabetes (29 kg/m^2^). The opposite was true for the average diastolic blood pressure, despite a similar systolic blood pressure across groups (*Table* 1).

Patients with diabetes receiving insulin more often had an ischaemic aetiology (45%) than either those with diabetes not on insulin (37%) or those without diabetes (35%). They were more likely to have undergone coronary revascularization than the latter two groups (39% vs. 27% and 19%, respectively). There was a similar difference in the prevalence of dyslipidaemia (75%, 62%, and 45% of each group, respectively) and the history of prior HF hospitalization (49%, 43%, and 40% of each group, respectively). Patients with diabetes, regardless of insulin use, had a higher prevalence of myocardial infarction, hypertension and stroke than patients without diabetes.

The use of loop diuretics was much more frequent in insulin‐treated patients (80%) than in those with diabetes not on insulin (66%) and especially compared to those without diabetes (56%), as was the use of lipid lowering agents (65%, 50%, and 35% of each group, respectively). There was also a significant difference in the use of mineralocorticoid receptor antagonists (30%, 23%, and 20% of each group, respectively) and of angiotensin‐converting enzyme inhibitors or angiotensin receptor blockers (76%, 71%, and 61%, respectively).

Although the mean LVEF differed little according to diabetes status or the use of insulin, patients treated with insulin had worse NYHA functional status, more HF‐related signs and symptoms, and worse health‐related quality of life, as indicated by a substantially higher MLWHF score and lower KCCQ clinical summary score compared to patients with diabetes not on insulin and, in particular, those without diabetes. For example, the proportion of patients with oedema was much higher in patients with insulin‐treated diabetes than in the other two groups (64%, 54%, and 48%, respectively).

The median plasma NT‐proBNP concentration was highest in insulin‐treated individuals (581 pg/mL) and lowest in those without diabetes (364 pg/mL), with an intermediate level in patients with diabetes not treated with insulin (430 pg/mL). There was a similar pattern in the proportion of patients with an eGFR < 60 mL/min/1.73 m^2^ (53%, 32%, and 37% of each group, respectively).

### Echocardiographic measurements

Of the 1398 patients with echocardiographic data available, 495 (35%) had diabetes at baseline. There were 206 (42%) diabetes patients on insulin treatment. The patients in this subset (and the differences among them in relation to diabetes status) were similar to those in the overall study cohort (online supplementary *Table* S6).

Wall thickness, left ventricular mass and the proportion of participants with left ventricular hypertrophy was higher among those with diabetes not taking insulin than in those without diabetes and highest in those with diabetes treated with insulin (*Table* 2). Although there was no difference in LVEF, fractional shortening and mitral lateral annular tissue velocity during systole (S′ lateral) were lower in diabetic patients on insulin than the other two groups. The higher early diastolic mitral inflow velocity (E) and E/E' ratio in diabetes patients treated with insulin, compared to the other patient groups, suggested insulin‐treated patients had higher left ventricular end‐diastolic pressure and more diastolic dysfunction than the other participants. However, left atrial size was lowest in diabetes patients treated with insulin compared to the other two groups.

**Table 2:** Echocardiographic data according to baseline diabetes mellitus status and insulin use

	Patients with data, *n* (%)	No DM (*n* = 903)	DM not on insulin (*n* = 289)	DM on insulin (*n* = 206)	*P*‐value
Age, years		72.1 ± 8.3	71.4 ± 8.4	68.8 ± 8.6	<0.0001
Male sex		370 (41.0)	156 (54.0)	98 (47.6)	0.0004
LV structure					
End‐diastolic dimension, cm	1291 (92)	4.8 ± 0.6	4.9 ± 0.6	4.8 ± 0.6	0.0139
End‐diastolic volume, mL	1176 (84)	92.3 ± 35.5	98.0 ± 34.6	101.1 ± 32.8	0.0032
End‐systolic dimension, cm	1267 (91)	3.2 ± 0.6	3.4 ± 0.5	3.4 ± 0.5	0.0003
End‐systolic volume, mL	1176 (84)	35.8 ± 19.0	38.5 ± 18.4	39.4 ± 16.4	0.0183
Septum wall thickness, cm	1331 (95)	1.06 ± 0.21	1.12 ± 0.22	1.21 ± 0.24	<0.0001
Posterior wall thickness, cm	1330 (95)	1.01 ± 0.20	1.07 ± 0.20	1.16 ± 0.22	<0.0001
Relative wall thickness	1283 (92)	0.44 ± 0.10	0.45 ± 0.11	0.49 ± 0.11	<0.0001
LV mass, g	1283 (92)	182.7 ± 65.4	204.8 ± 62.8	223.7 ± 72.1	<0.0001
LV mass index, g/m^2^	1278 (91)	95.7 ± 30.2	103.4 ± 28.2	106.4 ± 28.5	<0.0001
LV hypertrophy^a^	1278 (91)	268 (32.9)	107 (40.5)	98 (49.2)	<0.0001
LV systolic properties					
Fractional shortening, %	1258 (90)	32.6 ± 8.4	31.0 ± 7.1	30.8 ± 5.7	0.0013
Stroke volume, mL	1176 (84)	56.6 ± 21.3	59.4 ± 21.2	61.7 ± 19.8	0.0069
Ejection fraction, %	1234 (88)	61.9 ± 9.1	61.1 ± 8.8	60.9 ± 7.3	0.1929
S′ lateral, cm/s	889 (64)	7.8 ± 2.4	7.5 ± 2.4	7.2 ± 2.3	0.0222
LV diastolic properties					
E, cm/s	1289 (92)	80.1 ± 28.4	88.6 ± 29.9	99.8 ± 28.7	<0.0001
A, cm/s	1063 (76)	78.8 ± 26.5	79.0 ± 27.1	79.6 ± 27.2	0.9434
E/A	1057 (76)	1.6 ± 8.0	1.6 ± 4.7	1.8 ± 4.8	0.9686
E' lateral, cm/s	894 (64)	9.0 ± 3.6	8.7 ± 3.2	8.4 ± 3.1	0.1483
E' septal, cm/s	888 (64)	7.0 ± 3.0	6.6 ± 2.4	6.3 ± 2.2	0.0124
E/E' lateral	882 (63)	10.1 ± 4.7	11.4 ± 5.9	13.8 ± 6.0	<0.0001
E/E' septal	874 (63)	13.0 ± 5.9	15.4 ± 7.3	17.9 ± 7.4	<0.0001
E deceleration time, ms	1283 (92)	210.0 ± 74.0	210.4 ± 67.8	200.3 ± 53.6	0.2241
Isovolumic relaxation time, ms	636 (45)	96.5 ± 22.5	94.6 ± 19.9	87.5 ± 22.6	0.0295
Left atrial area, cm^2^	1267 (91)	21.8 ± 6.3	22.3 ± 6.0	20.6 ± 5.2	0.0144
Left atrial volume, mL	1091 (78)	74.2 ± 33.8	74.5 ± 33.7	63.7 ± 26.3	0.0006
Left atrial volume index, mL/m^2^	1084 (78)	39.5 ± 17.8	37.9 ± 17.6	31.2 ± 14.2	<0.0001

Values are given as *n* (%), or mean ± standard deviation.

A, peak late diastolic filling velocity during atrial contraction; DM, diabetes mellitus; E, peak early diastolic filling velocity; E', mitral lateral and septal annular tissue velocity during early filling; LV, left ventricular; S′ lateral, mitral lateral annular tissue velocity during systole.

a Defined as LV mass indexed to body surface area ≥ 115 g/m^2^ for men and ≥ 95 g/m^2^ for women.

### Clinical outcomes

**Figure 1:**
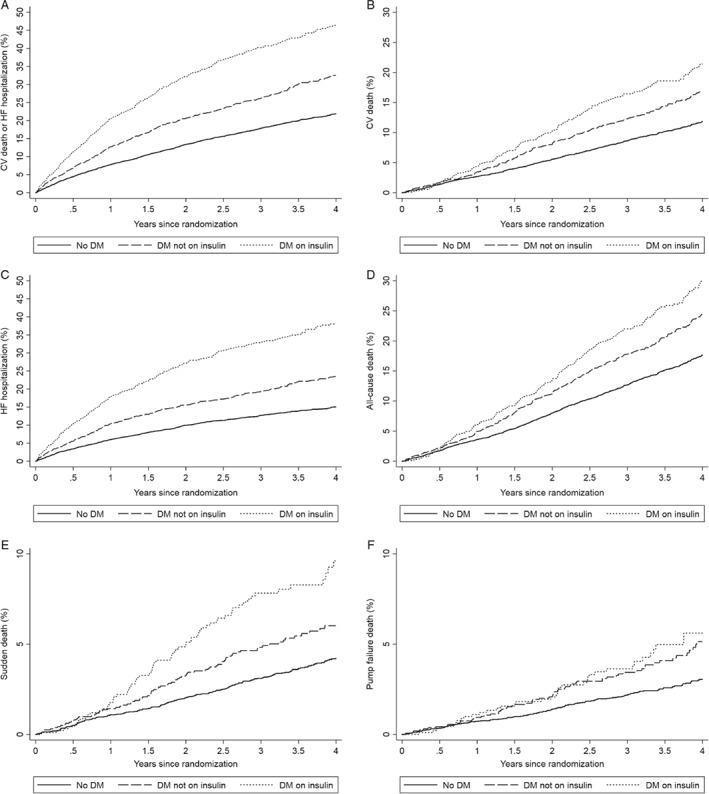
Kaplan–Meier curves for clinical outcomes according to baseline diabetes mellitus (DM) status and insulin use in the combined data sets of CHARM‐Preserved (left ventricular ejection fraction ≥ 45%), I‐Preserve and TOPCAT (Americas). Kaplan–Meier estimates of the probability of cardiovascular (CV) death or first hospitalization for heart failure (HF) (*A*), CV death (*B*), first hospitalization for HF (*C*), all‐cause death (*D*), sudden death (*E*) and pump failure death (*F*).

Incidence rates and HRs for the risk of each outcome of interest, according to diabetes status and insulin use at baseline, are presented in *Table* 3 and *Figure* 1.

**Table 3:** Clinical outcomes according to baseline diabetes mellitus status and insulin use in the combined data sets of CHARM‐Preserved (left ventricular ejection fraction ≥ 45%), I‐Preserve and TOPCAT (Americas)

	Patients, *n*	Event, *n* (%)	Annual rate per 100 person‐years (95% CI)	Unadjusted HR^a^ (95% CI)	Adjusted 1 HR^a^ (95% CI)	Adjusted 2 HR^a^ (95% CI)
CV death or HF hospitalization						
No DM	5813	1227 (21.1)	6.3 (6.0–6.7)	1.00 (Reference)	1.00 (Reference)	1.00 (Reference)
DM not on insulin	1674	513 (30.7)	10.2 (9.4–11.2)	1.58 (1.42–1.75), *P* < 0.001	1.53 (1.37–1.70), *P* < 0.001	1.49 (1.34–1.66), *P* < 0.001
DM on insulin	979	416 (42.5)	17.1 (15.5–18.8)	2.46 (2.20–2.76), *P* < 0.001	2.37 (2.10–2.67), *P* < 0.001	2.19 (1.94–2.48), *P* < 0.001
HF hospitalization						
No DM	5813	811 (14.0)	4.2 (3.9–4.5)			1.00 (Reference)
DM not on insulin	1674	354 (21.2)	7.1 (6.4–7.8)	1.62 (1.43–1.83), *P* < 0.001	1.54 (1.36–1.76), *P* < 0.001	1.50 (1.32–1.71), *P* < 0.001
DM on insulin	979	325 (33.2)	13.3 (12.0–14.9)	2.77 (2.43–3.16), *P* < 0.001	2.49 (2.16–2.87), *P* < 0.001	2.25 (1.95–2.60), *P* < 0.001
CV death						
No DM	5813	678 (11.7)	3.2 (3.0–3.5)	1.00 (Reference)	1.00 (Reference)	1.00 (Reference)
DM not on insulin	1674	259 (15.5)	4.6 (4.1–5.2)	1.42 (1.23–1.64), *P* < 0.001	1.45 (1.25–1.68), *P* < 0.001	1.43 (1.23–1.65), *P* < 0.001
DM on insulin	979	179 (18.3)	5.9 (5.1–6.8)	1.83 (1.55–2.17), *P* < 0.001	2.09 (1.75–2.50), *P* < 0.001	1.95 (1.63–2.33), *P* < 0.001
All‐cause death						
No DM	5813	1024 (17.6)	4.9 (4.6–5.2)	1.00 (Reference)	1.00 (Reference)	1.00 (Reference)
DM not on insulin	1674	387 (23.1)	6.8 (6.2–7.6)	1.39 (1.23–1.56), *P* < 0.001	1.44 (1.27–1.62), *P* < 0.001	1.41 (1.25–1.60), *P* < 0.001
DM on insulin	979	267 (27.3)	8.8 (7.8–9.9)	1.74 (1.52–2.00), *P* < 0.001	2.02 (1.75–2.34), *P* < 0.001	1.87 (1.61–2.16), *P* < 0.001
Sudden death						
No DM	5813	235 (4.0)	1.1 (1.0–1.3)	1.00 (Reference)	1.00 (Reference)	1.00 (Reference)
DM not on insulin	1674	93 (5.6)	1.6 (1.3–2.0)	1.49 (1.17–1.90), *P* = 0.001	1.46 (1.14–1.87), *P* = 0.003	1.46 (1.14–1.86), *P* = 0.003
DM on insulin	979	76 (7.8)	2.5 (2.0–3.1)	2.38 (1.83–3.10), *P* < 0.001	2.68 (2.03–3.54), *P* < 0.001	2.55 (1.93–3.37), *P* < 0.001
Pump failure death						
No DM	5813	158 (2.7)	0.8 (0.6–0.9)	1.00 (Reference)	1.00 (Reference)	1.00 (Reference)
DM not on insulin	1674	69 (4.1)	1.2 (1.0–1.5)	1.61 (1.21–2.13), *P* = 0.001	1.90 (1.42–2.55), *P* < 0.001	1.83 (1.36–2.46), *P* < 0.001
DM on insulin	979	41 (4.2)	1.4 (1.0–1.8)	1.72 (1.21–2.44), *P* = 0.002	2.38 (1.64–3.44), *P* < 0.001	2.14 (1.47–3.11), *P* < 0.001

BMI, body mass index; CI, confidence interval; CV, cardiovascular; DM, diabetes mellitus; eGFR, estimated glomerular filtration rate; HF, heart failure; HR, hazard ratio; LVEF, left ventricular ejection fraction; NT‐proBNP, N‐terminal pro‐B‐type natriuretic peptide; NYHA, New York Heart Association.

Adjustment Model 1: age, sex, heart rate, diastolic blood pressure, LVEF, NYHA class III/IV, BMI, HF hospitalization within the past 6 months, history of myocardial infarction, hypertension, and atrial fibrillation.

Adjustment Model 2: age, sex, race, heart rate, diastolic blood pressure, LVEF, NYHA class III/IV, BMI, HF hospitalization within the past 6 months, history of myocardial infarction, hypertension, and atrial fibrillation, eGFR, and log NT‐proBNP with simple imputation of eGFR and NT‐proBNP.

a HRs for combined data were adjusted for within‐trial clustering.

#### Composite outcomes and mortality

In unadjusted analyses, there was a stepwise increase in the rates of each of the primary composite outcome, HF hospitalization, CV death, and all‐cause death across the no diabetes (reference group), non‐insulin‐treated diabetes, and insulin‐treated diabetes groups. The rate of the primary composite outcome was 6.3 per 100 patient‐years in patients without diabetes, and 10.2 and 17.1 per 100 patient‐years in diabetes patients without and with insulin use, respectively. A similar magnitude of difference was seen in the rate of HF hospitalization (4.2, 7.1 and 13.3 per 100 patient‐years in each group, respectively). The elevated risks of these outcomes persisted after adjustment for the prognostic variables including NT‐proBNP. Compared to individuals without diabetes, there was a doubling in risk of the primary composite outcome in patients on insulin [fully adjusted HR 2.19; 95% confidence interval (CI) 1.94–2.48; *P* < 0.001], whereas patients with diabetes not treated with insulin had a 50% higher risk (HR 1.49; 95% CI 1.34–1.66; *P* < 0.001). The same was also true for the risk of HF hospitalization (fully adjusted HR 2.25; 95% CI 1.95–2.60; *P* < 0.001, and 1.50; 95% CI 1.32–1.71; *P* < 0.001, respectively) and, to a lesser extent, for CV death (HR 1.95; 95% CI 1.63–2.33; *P* < 0.001, and 1.43; 95% CI 1.23–1.65; *P* < 0.001, respectively) and for all‐cause death (HR 1.87; 95% CI 1.61–2.16; *P* < 0.001, and 1.41; 95% CI 1.25–1.60; *P* < 0.001, respectively).

#### Sudden death and pump failure death

In addition, we investigated the two main modes of CV death (*Table* 3). Compared with patients without diabetes, patients on insulin had a substantially higher risk of sudden death (fully adjusted HR 2.55; 95% CI 1.93–3.37; *P* < 0.001) as well as pump failure death (fully adjusted HR 2.14; 95% CI 1.47–3.11; *P* < 0.001). The elevated risks were attenuated, but remained significantly higher, in the other patients with diabetes not receiving insulin, with a fully adjusted HR of 1.46 (95% CI 1.14–1.86; *P* = 0.003) for sudden death and 1.83 (95% CI 1.36–2.46; *P* < 0.001) for pump failure death, respectively.

We also examined the outcomes of interest among patients with diabetes only (i.e. excluding those without diabetes in these comparisons) (*Table* 4). Overall, compared to those not receiving insulin, patients treated with insulin had significantly greater risks for the primary composite, its individual components, all‐cause death, and, particularly, sudden death. However, insulin use was not significantly associated with a higher risk of pump failure death. These associations were largely unchanged with adjustment for other prognostic variables including NT‐proBNP and the duration of diabetes.

**Table 4:** Clinical outcomes according to baseline insulin use in diabetic patients in the combined data sets of CHARM‐Preserved (left ventricular ejection fraction ≥ 45%), I‐Preserve and TOPCAT (Americas)

	Unadjusted HR^a^ (95% CI)	Adjusted 1 HR^a^ (95% CI)	Adjusted 2 HR^a^ (95% CI)	Adjusted 3 HR^a^ (95% CI)
CV death or HF hospitalization				
DM not on insulin	1.00 (Reference)	1.00 (Reference)	1.00 (Reference)	1.00 (Reference)
DM on insulin	1.57 (1.38–1.79), *P* < 0.001	1.51 (1.32–1.73), *P* < 0.001	1.43 (1.25–1.64), *P* < 0.001	1.41 (1.23–1.63), *P* < 0.001
HF hospitalization				
DM not on insulin	1.00 (Reference)	1.00 (Reference)	1.00 (Reference)	1.00 (Reference)
DM on insulin	1.72 (1.47–2.00), *P* < 0.001	1.58 (1.35–1.85), *P* < 0.001	1.46 (1.24–1.72), *P* < 0.001	1.45 (1.22–1.71), *P* < 0.001
CV death				
DM not on insulin	1.00 (Reference)	1.00 (Reference)	1.00 (Reference)	1.00 (Reference)
DM on insulin	1.32 (1.09–1.60), *P* = 0.005	1.42 (1.17–1.74), *P* = 0.001	1.35 (1.11–1.66), *P* = 0.003	1.32 (1.07–1.63), *P* = 0.009
All‐cause death				
DM not on insulin	1.00 (Reference)	1.00 (Reference)	1.00 (Reference)	1.00 (Reference)
DM on insulin	1.29 (1.10–1.51), *P* = 0.002	1.38 (1.18–1.63), *P* < 0.001	1.30 (1.11–1.54), *P* = 0.002	1.27 (1.07–1.50), *P* = 0.007
Sudden death				
DM not on insulin	1.00 (Reference)	1.00 (Reference)	1.00 (Reference)	1.00 (Reference)
DM on insulin	1.59 (1.17–2.16), *P* = 0.003	1.81 (1.32–2.49), *P* < 0.001	1.76 (1.28–2.42), *P* = 0.001	1.67 (1.20–2.32), *P* = 0.002
Pump failure death				
DM not on insulin	1.00 (Reference)	1.00 (Reference)	1.00 (Reference)	1.00 (Reference)
DM on insulin	1.13 (0.76–1.67), *P* = 0.537	1.30 (0.86–1.94), *P* = 0.209	1.18 (0.78–1.78), *P* = 0.429	1.20 (0.78–1.83), *P* = 0.411

BMI, body mass index; CI, confidence interval; CV, cardiovascular; DM, diabetes mellitus; eGFR, estimated glomerular filtration rate; HF, heart failure; HR, hazard ratio; LVEF, left ventricular ejection fraction; NT‐proBNP, N‐terminal pro‐B‐type natriuretic peptide; NYHA, New York Heart Association.

Adjustment Model 1: age, sex, heart rate, diastolic blood pressure, LVEF, NYHA class III/IV, BMI, HF hospitalization within the past 6 months, history of myocardial infarction, hypertension, and atrial fibrillation.

Adjustment Model 2: age, sex, heart rate, diastolic blood pressure, LVEF, NYHA class III/IV, BMI, HF hospitalization within the past 6 months, history of myocardial infarction, hypertension, and atrial fibrillation, eGFR, and log NT‐proBNP with simple imputation of eGFR and NT‐proBNP.

Adjustment Model 3: age, sex, race, heart rate, diastolic blood pressure, LVEF, NYHA class III/IV, BMI, HF hospitalization within the past 6 months, history of myocardial infarction, hypertension, and atrial fibrillation, eGFR, log NT‐proBNP and log diabetes duration with simple imputation of eGFR, NT‐proBNP and diabetes duration.

a HRs for combined data were adjusted for within‐trial clustering.

We conducted a number of sensitivity analyses, including exclusion of patients with age < 20 years at diabetes onset and examination of only the subset of patients with echocardiographic measures (online supplementary *Tables* S2–S5 and S7). None of these additional analyses resulted in any major change to the overall findings.

## Discussion

Previously we showed that, among patients with concurrent HFrEF and diabetes, those treated with insulin have worse symptoms, more signs of congestion and greater risks of death and HF hospitalization compared with those not on insulin.^14^ We believe our present report is the first to extend these observations to patients with HFpEF, and we have also identified additional, novel findings in this HF phenotype.

We found that HFpEF patients with diabetes, treated with insulin, had a higher median NT‐proBNP and evidence of more congestion than those not treated with insulin, despite their younger age and similar average LVEF and duration of HF. In keeping with prior studies, we found that patients with diabetes had more echocardiographic left ventricular hypertrophy than those without and this was especially true for patients treated with insulin. In keeping with this, elevated early diastolic mitral inflow velocity (E) and E/E' ratio suggested greater diastolic dysfunction and increased left ventricular end‐diastolic pressure in patients treated with insulin. Although these individuals also had higher NT‐proBNP levels (as described above), consistent with this hypothesis, they had smaller left atrial size. This apparent paradox may be explained by the lower prevalence of atrial fibrillation in patients treated with insulin, which itself is an interesting finding that has been highlighted recently.^21^ Tan and colleagues suggested that differences in cardiac remodelling might explain the relative ‘protection’ HFpEF patients with diabetes have against atrial fibrillation, although our data suggest that this difference is confined to patients treated with insulin.

We also found that HFpEF patients with insulin‐treated diabetes had worse health‐related quality of life and were twice as likely to experience CV death or HF hospitalization, even after extensive adjustment for other prognostic variables including NT‐proBNP and eGFR. These patients were at greater risk than not only individuals without diabetes but also those with diabetes treated with other glucose‐lowering agents.

The importance of measuring patient‐reported outcomes in HF is now widely recognized and in the trials included in our analyses the two most commonly used instruments, i.e. the MLWHF score and the KCCQ, were employed.^22^, ^23^ We found that patients with diabetes on insulin had a substantially worse quality of life, with the average value of the MLWHF score 6 and 11 (out of 105) points higher (worse) compared to patients with diabetes not on insulin and those without diabetes, respectively. Likewise, a similar magnitude of incremental worsening of quality of life was observed across groups when examined using the KCCQ clinical summary score (6 and 13 points out of 100 worse, respectively). As well as evidence of greater congestion, the worse health‐related quality of life may reflect higher BMI, more coronary heart disease and greater renal impairment.^24^, ^25^ It is particularly notable that, whereas patient‐reported outcomes were markedly different among the groups examined, NYHA class (a physician‐reported outcome) was not. This suggests that physicians may not fully appreciate the impact of diabetes on health‐related quality of life in patients with HFpEF and reinforces the value of using patient‐reported outcomes.

In addition to worse patient‐reported outcomes, individuals with diabetes, especially those treated with insulin, had worse clinical outcomes compared to patients without diabetes. Although a few studies have examined outcomes in HFpEF patients according to diabetes status, individually they have been too small to compare insulin‐treated patients to those not receiving insulin, could not adjust for all other important prognostic markers (particularly natriuretic peptides), or had both limitations.^26^ We found that, even after adjustment for NT‐proBNP and other key prognostic variables, use of insulin to treat diabetes was associated with around a doubling in risks for the composite of HF hospitalization or CV death, its individual components, and all‐cause mortality, and a 50% higher risk of these outcomes when other glucose‐lowering therapies were employed.

Another strength of our study was that it was large enough to examine individual modes of death and these were adjudicated. Notably, we found that the greater risk of CV death was due to a higher rate of sudden death rather than pump failure death in insulin‐treated compared to non‐insulin‐treated patients with diabetes. This was an unexpected finding. A higher risk for pump failure rather than sudden death might have been predicted by the greater severity of symptoms and signs of congestion in insulin‐treated patients. It is possible, however, to postulate mechanisms leading to a greater risk of sudden death. The first relates to diabetes *per se* and, particularly, the development of autonomic neuropathy, which can cause cardiac electrical instability. The second relates to co‐morbidity, especially coronary artery disease which may predispose to ventricular arrhythmias. Thirdly, insulin treatment and the associated risk of hypoglycaemia may also be relevant. Hypoglycaemia has several adverse CV effects such as adrenergic activation, tachycardia, excessive compensatory vagal activation, bradycardia, myocardial ischaemia, hypokalaemia, and QT interval prolongation, all potentially leading to lethal arrhythmias.^12^, ^27^, ^28^ Insulin‐treated patients also have more adverse cardiac remodelling. Of course, these possible mechanisms are not mutually exclusive and are potentially additive.

What are the clinical implications of our study? Patients with insulin‐treated diabetes have more congestion and physicians should be vigilant for this and consider appropriate use of diuretics to try to achieve euvolaemia. This may also help ameliorate the substantially worse health‐related quality of life in patients with insulin‐treated diabetes. More controversial is what to do about the initiation of insulin in patients with diabetes. Insulin has the advantage of being effective when combination therapy with other agents fails to achieve the glycaemic goal and improved glycaemic control can protect against microvascular complications that may lead to blindness and renal failure. However, this advantage may be accompanied by an increase in hypoglycaemia episodes and in weight. In addition, our findings showed insulin is associated with worse clinical outcomes in HF, although this is yet to be tested in a prospective randomized controlled trial, which is the only way of determining whether insulin is safe in HF. In the absence of such a trial, it may be preferable to avoid insulin, if possible, especially as there is some evidence that an alternative class of glucose‐lowering agents, the SGLT2 inhibitors, may be safe in HF.^7^, ^8^, ^9^, ^10^ However, prospective randomized outcome trials with these drugs in patients with established HF are required and are now underway.^29^

Our study has several limitations. The most important is that insulin treatment was not randomized and patients on insulin had longer standing diabetes, implying a greater severity of diabetes, and in turn, as indicated above, there were substantial differences in baseline characteristics between patients treated with and without insulin. While we attempted to adjust for these differences in our multivariable analyses, unmeasured confounders could not be accounted for.^30^ In one large placebo‐controlled trial in patients with pre‐diabetes and established type 2 diabetes, insulin did not increase the risk of incident or recurrent HF hospitalizations, although patients in NYHA class III or IV were not enrolled in this trial and the prevalence of HF at baseline was not recorded.^31^, ^32^

Several other limitations should also be acknowledged. First, our study was not prospectively planned, i.e. was retrospective. Second, the diagnosis of diabetes was reported by investigators without systematic documentation using standardized diagnostic criteria. It is possible that some cases were missed. Previous studies have identified that 8–22% of HF patients have undiagnosed diabetes.^33^, ^34^, ^35^ However, the misclassification of diabetes, if any, would weaken rather than strengthen effect estimates. Third, there was no differentiation between type 1 and type 2 diabetes. However, the overall findings did not change in the sensitivity analysis with the exclusion of patients having diabetes diagnosed under 20 years of age (4.4%), who were presumably type 1 diabetes (online supplementary *Tables* S1–S5 and *Figure* S1). Fourth, the TOPCAT Americas data have been published separately, although these account for only 21% of the total patients in the present data set in which we have conducted more comprehensive analyses.^15^ Finally, data on the type and dose of insulin (and other glucose‐lowering therapies), the level of glycaemic control (e.g. measurement of glycated haemoglobin), and burden of microvascular complications in patients with diabetes were not available. The trials included in this individual‐patient pooled analysis were conducted before the introduction of novel glucose‐lowering therapies including dipeptidyl peptidase‐4 and SGLT2 inhibitors and glucagon‐like peptide‐1 receptor agonists.

In conclusion, among patients with HFpEF, those with diabetes taking insulin have more evidence of congestion, worse health‐related quality of life, higher concentrations of NT‐proBNP, and worse clinical outcomes, including higher risks of HF hospitalization, CV death, and all‐cause death, as compared to those not treated with insulin and, particularly, individuals without diabetes. The excess mortality in insulin‐treated patients is driven by a higher risk of sudden death. Whether the association between insulin use and poor outcomes in HFpEF is causal should be investigated in a prospective randomized controlled trial.

### Funding

J.J.V.McM. is supported by British Heart Foundation Centre of Excellence award RE/18/6/34217.

**Conflict of interest**: none declared.

## Supporting information


**Table S1.** Baseline characteristics according to baseline diabetes mellitus status and insulin use in the combined data sets of CHARM‐Preserved (LVEF ≥ 45%), I‐Preserve and TOPCAT (Americas) after excluding patients with age < 20 years at diabetes onset.
**Table S2.** Clinical outcomes according to baseline diabetes mellitus status and insulin use in the combined data sets of CHARM‐Preserved (LVEF ≥ 45%), I‐Preserve and TOPCAT (Americas) after excluding patients with age < 20 years at diabetes onset.
**Table S3.** Clinical outcomes according to baseline insulin use in diabetic patients in the combined data sets of CHARM‐Preserved (LVEF ≥ 45%), I‐Preserve and TOPCAT (Americas) after excluding patients with age < 20 years at diabetes onset.
**Table S4.** Clinical outcomes according to baseline diabetes mellitus status and insulin use in the combined data sets of CHARM‐Preserved (LVEF ≥ 45%), I‐Preserve and TOPCAT (Americas) in patients with NT‐proBNP available after excluding patients with age < 20 years at diabetes onset.
**Table S5.** Clinical outcomes according to baseline insulin use in diabetic patients in the combined data sets of CHARM‐Preserved (LVEF ≥ 45%), I‐Preserve and TOPCAT (Americas) in patients with NT‐proBNP available after excluding patients with age < 20 years at diabetes onset.
**Table S6.** Baseline characteristics according to baseline diabetes mellitus status and insulin use among patients with full echocardiographic examination.
**Table S7.** Clinical outcomes according to baseline diabetes mellitus status and insulin use in the combined data sets of CHARM‐Preserved (LVEF ≥ 45%), I‐Preserve and TOPCAT (Americas) (only patients with echocardiographic data).
**Figure S1.** Cumulative incidences for clinical outcomes according to baseline diabetes mellitus status and insulin use in the combined data sets of CHARM‐Preserved (LVEF ≥ 45%), I‐Preserve and TOPCAT (Americas) after excluding patients with age < 20 years at diabetes onset.

## References

[1] Solomon SD , Rizkala AR , Lefkowitz MP , Shi VC , Gong J , Anavekar N , Anker SD , Arango JL , Arenas JL , Atar D , Ben‐Gal T , Boytsov SA , Chen CH , Chopra VK , Cleland J , Comin‐Colet J , Duengen HD , Echeverria Correa LE , Filippatos G , Flammer AJ , Galinier M , Godoy A , Goncalvesova E , Janssens S , Katova T , Kober L , Lelonek M , Linssen G , Lund LH , O'Meara E , Merkely B , Milicic D , Oh BH , Perrone SV , Ranjith N , Saito Y , Saraiva JF , Shah S , Seferovic PM , Senni M , Sibulo AS Jr , Sim D , Sweitzer NK , Taurio J , Vinereanu D , Vrtovec B , Widimsky J Jr , Yilmaz MB , Zhou J , Zweiker R , Anand IS , Ge J , Lam CS , Maggioni AP , Martinez F , Packer M , Pfeffer MA , Pieske B , Redfield MM , Rouleau JL , Van Veldhuisen DJ , Zannad F , Zile MR , McMurray JJ . Baseline characteristics of patients with heart failure and preserved ejection fraction in the PARAGON‐HF trial. Circ Heart Fail 2018;11:e004962. 29980595 10.1161/CIRCHEARTFAILURE.118.004962

[2] Kristensen SL , Mogensen UM , Jhund PS , Petrie MC , Preiss D , Win S , Kober L , McKelvie RS , Zile MR , Anand IS , Komajda M , Gottdiener JS , Carson PE , McMurray JJ . Clinical and echocardiographic characteristics and cardiovascular outcomes according to diabetes status in patients with heart failure and preserved ejection fraction: a report from the I‐Preserve trial (Irbesartan in Heart Failure with Preserved Ejection Fraction). Circulation 2017;135:724–735. 28052977 10.1161/CIRCULATIONAHA.116.024593

[3] MacDonald MR , Petrie MC , Varyani F , Ostergren J , Michelson EL , Young JB , Solomon SD , Granger CB , Swedberg K , Yusuf S , Pfeffer MA , McMurray JJ ; CHARM Investigators. Impact of diabetes on outcomes in patients with low and preserved ejection fraction heart failure: an analysis of the Candesartan in Heart failure: Assessment of Reduction in Mortality and morbidity (CHARM) programme. Eur Heart J 2008;29:1377–1385. 18413309 10.1093/eurheartj/ehn153

[4] Papp Z , Radovits T , Paulus WJ , Hamdani N , Seferovic PM . Molecular and pathophysiological links between heart failure with preserved ejection fraction and type 2 diabetes mellitus. Eur J Heart Fail 2018;20:1649–1652. 30280460 10.1002/ejhf.1318

[5] Scirica BM , Braunwald E , Raz I , Cavender MA , Morrow DA , Jarolim P , Udell JA , Mosenzon O , Im K , Umez‐Eronini AA , Pollack PS , Hirshberg B , Frederich R , Lewis BS , McGuire DK , Davidson J , Steg PG , Bhatt DL ; SAVOR‐TIMI 53 Steering Committee and Investigators. Heart failure, saxagliptin, and diabetes mellitus: observations from the SAVOR‐TIMI 53 randomized trial. Circulation 2014;130:1579–1588. 25189213 10.1161/CIRCULATIONAHA.114.010389

[6] Lago RM , Singh PP , Nesto RW . Congestive heart failure and cardiovascular death in patients with prediabetes and type 2 diabetes given thiazolidinediones: a meta‐analysis of randomised clinical trials. Lancet 2007;370:1129–1136. 17905165 10.1016/S0140-6736(07)61514-1

[7] Kramer CK , Ye C , Campbell S , Retnakaran R . Comparison of new glucose‐lowering drugs on risk of heart failure in type 2 diabetes: a network meta‐analysis. JACC Heart Fail 2018;6:823–830. 30196071 10.1016/j.jchf.2018.05.021

[8] Zelniker TA , Wiviott SD , Raz I , Im K , Goodrich EL , Furtado RH , Bonaca MP , Mosenzon O , Kato ET , Cahn A , Bhatt DL , Leiter LA , McGuire DK , Wilding JP , Sabatine MS . Comparison of the effects of glucagon‐like peptide receptor agonists and sodium‐glucose co‐transporter 2 inhibitors for prevention of major adverse cardiovascular and renal outcomes in type 2 diabetes mellitus: a systematic review and meta‐analysis of cardiovascular outcomes trials. Circulation 2019;139:2022–2031. 30786725 10.1161/CIRCULATIONAHA.118.038868

[9] Zelniker TA , Wiviott SD , Raz I , Im K , Goodrich EL , Bonaca MP , Mosenzon O , Kato ET , Cahn A , Furtado RH , Bhatt DL , Leiter LA , McGuire DK , Wilding JP , Sabatine MS . SGLT2 inhibitors for primary and secondary prevention of cardiovascular and renal outcomes in type 2 diabetes: a systematic review and meta‐analysis of cardiovascular outcome trials. Lancet 2019;393:31–39. 30424892 10.1016/S0140-6736(18)32590-X

[10] Hernandez AF , Green JB , Janmohamed S , D'Agostino RB , Granger CB , Jones NP , Leiter LA , Rosenberg AE , Sigmon KN , Somerville MC , Thorpe KM , McMurray JJ , Del Prato S ; Harmony Outcomes Committees and Investigators. Albiglutide and cardiovascular outcomes in patients with type 2 diabetes and cardiovascular disease (Harmony Outcomes): a double‐blind, randomised placebo‐controlled trial. Lancet 2018;392:1519–1529. 30291013 10.1016/S0140-6736(18)32261-X

[11] O'Brien MJ , Karam SL , Wallia A , Kang RH , Cooper AJ , Lancki N , Moran MR , Liss DT , Prospect TA , Ackermann RT . Association of second‐line antidiabetic medications with cardiovascular events among insured adults with type 2 diabetes. JAMA Netw Open 2018;1:e186125. 10.1001/jamanetworkopen.2018.6125 PMC6324353 30646315

[12] Davis SN , Duckworth W , Emanuele N , Hayward RA , Wiitala WL , Thottapurathu L , Reda DJ , Reaven PD ; Investigators of the Veterans Affairs Diabetes Trial.Effects of severe hypoglycemia on cardiovascular outcomes and death in the Veterans Affairs Diabetes Trial. Diabetes Care 2019;42:157–163. 30455335 10.2337/dc18-1144 PMC6463547

[13] Pocock SJ , Wang D , Pfeffer MA , Yusuf S , McMurray JJ , Swedberg KB , Östergren J , Michelson EL , Pieper KS , Granger CB . Predictors of mortality and morbidity in patients with chronic heart failure. Eur Heart J 2006;27:65–75. 16219658 10.1093/eurheartj/ehi555

[14] Cosmi F , Shen L , Magnoli M , Abraham WT , Anand IS , Cleland JG , Cohn JN , Cosmi D , De Berardis G , Dickstein K , Franzosi MG , Gullestad L , Jhund PS , Kjekshus J , Kober L , Lepore V , Lucisano G , Maggioni AP , Masson S , McMurray JJ , Nicolucci A , Petrarolo V , Robusto F , Staszewsky L , Tavazzi L , Teli R , Tognoni G , Wikstrand J , Latini R . Treatment with insulin is associated with worse outcome in patients with chronic heart failure and diabetes. Eur J Heart Fail 2018;20:888–895. 29488676 10.1002/ejhf.1146

[15] Huynh T , Harty BJ , Claggett B , Fleg JL , McKinlay SM , Anand IS , Lewis EF , Joseph J , Desai AS , Sweitzer NK , EileenO'Meara PB , Pfeffer MA , Rouleau JL . Comparison of outcomes in patients with diabetes mellitus treated with versus without insulin + heart failure with preserved left ventricular ejection fraction (from the TOPCAT study). Am J Cardiol 2019;123:611–617. 30612727 10.1016/j.amjcard.2018.11.022 PMC6349530

[16] Paulus WJ , Dal Canto E . Distinct myocardial targets for diabetes therapy in heart failure with preserved or reduced ejection fraction. JACC Heart Fail 2018;6:1–7. 29284577 10.1016/j.jchf.2017.07.012

[17] Yusuf S , Pfeffer MA , Swedberg K , Granger CB , Held P , McMurray JJ , Michelson EL , Olofsson B , Östergren J . Effects of candesartan in patients with chronic heart failure and preserved left‐ventricular ejection fraction: the CHARM‐Preserved trial. Lancet 2003;362:777–781. 13678871 10.1016/S0140-6736(03)14285-7

[18] Massie BM , Carson PE , McMurray JJ , Komajda M , McKelvie R , Zile MR , Anderson S , Donovan M , Iverson E , Staiger C , Ptaszynska A ; I‐PRESERVE Investigators. Irbesartan in patients with heart failure and preserved ejection fraction. N Engl J Med 2008;359:2456–2467. 19001508 10.1056/NEJMoa0805450

[19] Pitt B , Pfeffer MA , Assmann SF , Boineau R , Anand IS , Claggett B , Clausell N , Desai AS , Diaz R , Fleg JL , Gordeev I , Harty B , Heitner JF , Kenwood CT , Lewis EF , O'Meara E , Probstfield JL , Shaburishvili T , Shah SJ , Solomon SD , Sweitzer NK , Yang S , McKinlay SM ; TOPCAT Investigators. Spironolactone for heart failure with preserved ejection fraction. N Engl J Med 2014;370:1383–1392. 24716680 10.1056/NEJMoa1313731

[20] Pfeffer MA , Claggett B , Assmann SF , Boineau R , Anand IS , Clausell N , Desai AS , Diaz R , Fleg JL , Gordeev I , Heitner JF , Lewis EF , O'Meara E , Rouleau JL , Probstfield JL , Shaburishvili T , Shah SJ , Solomon SD , Sweitzer NK , McKinlay SM , Pitt B . Regional variation in patients and outcomes in the Treatment of Preserved Cardiac Function Heart Failure With an Aldosterone Antagonist (TOPCAT) trial. Circulation 2015;131:34–42. 25406305 10.1161/CIRCULATIONAHA.114.013255

[21] Tan ES , Tay WT , Teng TK , Sim D , Leong KT , Yeo PS , Ong HY , Jaufeerally F , Ng TP , Poppe K , Lund M , Devlin G , Troughton RW , Ling LH , Richards AM , Doughty RN , Lam CS . Ethnic differences in atrial fibrillation in patients with heart failure from Asia‐Pacific. Heart 2019;105:842–847. 30661038 10.1136/heartjnl-2018-314077

[22] U.S. Department of Health and Human Services FDA Center for Drug Evaluation and Research; U.S. Department of Health and Human Services FDA Center for Biologics Evaluation and Research; U.S. Department of Health and Human Services FDA Center for Devices and Radiological Health . Guidance for industry: patient‐reported outcome measures: use in medical product development to support labeling claims: draft guidance. Health Qual Life Outcomes 2006;4:79. 17034633 10.1186/1477-7525-4-79 PMC1629006

[23] Napier R , McNulty SE , Eton DT , Redfield MM , AbouEzzeddine O , Dunlay SM . Comparing measures to assess health‐related quality of life in heart failure with preserved ejection fraction. JACC Heart Fail 2018;6:552–560. 29885952 10.1016/j.jchf.2018.02.006 PMC6026057

[24] Lewis EF , Lamas GA , O'Meara E , Granger CB , Dunlap ME , McKelvie RS , Probstfield JL , Young JB , Michelson EL , Halling K , Carlsson J , Olofsson B , McMurray JJ , Yusuf S , Swedberg K , Pfeffer MA ; CHARM Investigators. Characterization of health‐related quality of life in heart failure patients with preserved versus low ejection fraction in CHARM. Eur J Heart Fail 2007;9:83–91. 17188020 10.1016/j.ejheart.2006.10.012

[25] Carson P , Tam SW , Ghali JK , Archambault WT , Taylor A , Cohn JN , Braman VM , Worcel M , Anand IS . Relationship of quality of life scores with baseline characteristics and outcomes in the African‐American heart failure trial. J Card Fail 2009;15:835–842. 19944359 10.1016/j.cardfail.2009.05.016

[26] Sandesara PB , O'Neal WT , Kelli HM , Samman‐Tahhan A , Hammadah M , Quyyumi AA , Sperling LS . The prognostic significance of diabetes and microvascular complications in patients with heart failure with preserved ejection fraction. Diabetes Care 2018;41:150–155. 29051160 10.2337/dc17-0755 PMC5741155

[27] Hanefeld M , Frier BM , Pistrosch F . Hypoglycemia and cardiovascular risk: is there a major link? Diabetes Care 2016;39:S205–S209. 27440834 10.2337/dcS15-3014

[28] Chow E , Bernjak A , Williams S , Fawdry RA , Hibbert S , Freeman J , Sheridan PJ , Heller SR . Risk of cardiac arrhythmias during hypoglycemia in patients with type 2 diabetes and cardiovascular risk. Diabetes 2014;63:1738–1747. 24757202 10.2337/db13-0468

[29] McMurray JJ , DeMets DL , Inzucchi SE , Kober L , Kosiborod MN , Langkilde AM , Martinez FA , Bengtsson O , Ponikowski P , Sabatine MS , Sjostrand M , Solomon SD ; DAPA‐HF Committees and Investigators. A trial to evaluate the effect of the sodium‐glucose co‐transporter 2 inhibitor dapagliflozin on morbidity and mortality in patients with heart failure and reduced left ventricular ejection fraction (DAPA‐HF). Eur J Heart Fail 2019;21:665–675. 30895697 10.1002/ejhf.1432 PMC6607736

[30] Gerstein HC , McMurray J , Holman RR . Real‐world studies no substitute for RCTs in establishing efficacy. Lancet 2019;393:210–211. 30663582 10.1016/S0140-6736(18)32840-X

[31] Investigators OT , Gerstein HC , Bosch J , Dagenais GR , Diaz R , Jung H , Maggioni AP , Pogue J , Probstfield J , Ramachandran A , Riddle MC , Ryden LE , Yusuf S ; ORIGIN Trial Investigators. Basal insulin and cardiovascular and other outcomes in dysglycemia. N Engl J Med 2012;367:319–328. 22686416 10.1056/NEJMoa1203858

[32] Gerstein HC , Jung H , Ryden L , Diaz R , Gilbert RE , Yusuf S ; ORIGIN Investigators.Effect of basal insulin glargine on first and recurrent episodes of heart failure hospitalization: the ORIGIN trial (Outcome Reduction With Initial Glargine Intervention). Circulation 2018;137:88–90. 29279340 10.1161/CIRCULATIONAHA.117.030924

[33] Kristensen SL , Preiss D , Jhund PS , Squire I , Cardoso JS , Merkely B , Martinez F , Starling RC , Desai AS , Lefkowitz MP , Rizkala AR , Rouleau JL , Shi VC , Solomon SD , Swedberg K , Zile MR , McMurray JJ , Packer M ; PARADIGM‐HF Investigators and Committees. Risk related to pre‐diabetes mellitus and diabetes mellitus in heart failure with reduced ejection fraction: insights from Prospective Comparison of ARNI with ACEI to Determine Impact on Global Mortality and Morbidity in Heart Failure trial. Circ Heart Fail 2016;9:e002560. 26754626 10.1161/CIRCHEARTFAILURE.115.002560 PMC4718182

[34] Suskin N , McKelvie RS , Burns RJ , Latini R , Pericak D , Probstfield J , Rouleau JL , Sigouin C , Solymoss CB , Tsuyuki R , White M , Yusuf S . Glucose and insulin abnormalities relate to functional capacity in patients with congestive heart failure. Eur Heart J 2000;21:1368–1375. 10952826 10.1053/euhj.1999.2043

[35] Kristensen SL , Jhund PS , Lee MM , Kober L , Solomon SD , Granger CB , Yusuf S , Pfeffer MA , Swedberg K , McMurray JJ ; CHARM Investigators and Committees. Prevalence of prediabetes and undiagnosed diabetes in patients with HFpEF and HFrEF and associated clinical outcomes. Cardiovasc Drugs Ther 2017;31:545–549. 28948430 10.1007/s10557-017-6754-x PMC5730631

