# Supplementary material for: Insulin treatment and clinical outcomes in patients with diabetes and heart failure with preserved ejection fraction
Source: Eur J Heart Fail. 2019 Jul 4;21(8):974–84. doi: 10.1002/ejhf.1535 (PMC7079555; doi:10.1002/ejhf.1535)
Supplement: Supplementary file 1 — Table S1. Baseline characteristics according to baseline diabetes mellitus status and insulin use in the combined data sets of CHARM‐Preserved (LVEF ≥ 45%), I‐Preserve and TOPCAT (Americas) after excluding patients with age < 20 years at diabetes onset. Table S2. Clinical outcomes according to baseline diabetes mellitus status and insulin use in the combined data sets of CHARM‐Preserved (LVEF ≥ 45%), I‐Preserve and TOPCAT (Americas) after excluding patients with age < 20 years at diabetes onset. Table S3. Clinical outcomes according to baseline insulin use in diabetic patients in the combined data sets of CHARM‐Preserved (LVEF ≥ 45%), I‐Preserve and TOPCAT (Americas) after excluding patients with age < 20 years at diabetes onset. Table S4. Clinical outcomes according to baseline diabetes mellitus status and insulin use in the combined data sets of CHARM‐Preserved (LVEF ≥ 45%), I‐Preserve and TOPCAT (Americas) in patients with NT‐proBNP available after excluding patients with age < 20 years at diabetes onset. Table S5. Clinical outcomes according to baseline insulin use in diabetic patients in the combined data sets of CHARM‐Preserved (LVEF ≥ 45%), I‐Preserve and TOPCAT (Americas) in patients with NT‐proBNP available after excluding patients with age < 20 years at diabetes onset. Table S6. Baseline characteristics according to baseline diabetes mellitus status and insulin use among patients with full echocardiographic examination. Table S7. Clinical outcomes according to baseline diabetes mellitus status and insulin use in the combined data sets of CHARM‐Preserved (LVEF ≥ 45%), I‐Preserve and TOPCAT (Americas) (only patients with echocardiographic data). Figure S1. Cumulative incidences for clinical outcomes according to baseline diabetes mellitus status and insulin use in the combined data sets of CHARM‐Preserved (LVEF ≥ 45%), I‐Preserve and TOPCAT (Americas) after excluding patients with age < 20 years at diabetes onset. [file EJHF-21-974-s001.docx]

**Supplementary analyses**

Table S1. Baseline characteristics according to baseline diabetes mellitus status and insulin use in the combined data sets of CHARM-Preserved (LVEF ≥45%), I-Preserve and TOPCAT (Americas) **after excluding patients with age at diabetes onset below 20 years**

Table S2. Clinical outcomes according to baseline diabetes mellitus status and insulin use in the combined data sets of CHARM-Preserved (LVEF ≥45%), I-Preserve and TOPCAT (Americas) **after excluding patients with age at diabetes onset below 20 years**

Table S3. Clinical outcomes according to baseline insulin use in diabetic patients in the combined data sets of CHARM-Preserved (LVEF ≥45%), I-Preserve and TOPCAT (Americas) **after excluding patients with age at diabetes onset below 20 years**

Table S4. Clinical outcomes according to baseline diabetes mellitus status and insulin use in the combined data sets of CHARM-Preserved (LVEF ≥45%), I-Preserve and TOPCAT (Americas) **in patients with NT-proBNP available** **after excluding patients with age at diabetes onset below 20 years**

Table S5. Clinical outcomes according to baseline insulin use in diabetic patients in the combined data sets of CHARM-Preserved (LVEF ≥45%), I-Preserve and TOPCAT (Americas) **in patients with NT-proBNP available** **after excluding patients with age at diabetes onset below 20 years**

Table S6. Baseline characteristics according to baseline diabetes mellitus status and insulin use among patients with full echocardiographic examination

Table S7. Clinical outcomes according to baseline diabetes mellitus status and insulin use in the combined data sets of CHARM-Preserved (LVEF ≥45%), I-Preserve and TOPCAT (Americas) (Only Patients with Echocardiographic Data).

Figure S1. Cumulative incidences for clinical outcomes according to baseline diabetes mellitus status and insulin use in the combined data sets of CHARM-Preserved (LVEF ≥45%), I-Preserve and TOPCAT (Americas) **after excluding patients with age at diabetes onset below 20 years**

Cumulative incidences of the CV death or first hospitalization for HF (A), CV death (B), first hospitalization for HF (C), death from any cause (D), sudden death (E) and pump failure death (F). CV indicates cardiovascular; and HF, heart failure.

Table S1. Baseline characteristics according to baseline diabetes mellitus status and insulin use in the combined data sets of CHARM-Preserved (LVEF ≥45%), I-Preserve and TOPCAT (Americas) **after excluding patients with age at diabetes onset below 20 years**

|  | No DM | DM not on insulin | DM on insulin | p value | | | |
| --- | --- | --- | --- | --- | --- | --- | --- |
|  | n=5813 | n=1643 | n=944 | overall | DM not on insulin vs. no DM | DM on insulin vs. no DM | DM on insulin vs. not on insulin |
| Age -years | 70.6 ± 9.4 | 69.9 ± 8.7 | 68.0 ± 8.2 | <0.0001 | 0.011 | <0.001 | <0.001 |
| Age at diabetes onset -years |  | 59.7 ± 11.5 | 50.4 ± 12.0 |  |  |  | <0.0001 |
| Duration of diabetes -years |  | 7 (3-12) | 15 (9-22) |  |  |  | <0.0001 |
| Male sex, n (%) | 2724 (46.9) | 820 (49.9) | 435 (46.1) | 0.0645 | 0.029 | 0.656 | 0.061 |
| Race, n (%) |  |  |  | <0.0001 | <0.001 | <0.001 | 0.001 |
| White | 5357 (92.2) | 1403 (85.4) | 782 (82.8) |  |  |  |  |
| Black | 248 (4.3) | 122 (7.4) | 111 (11.8) |  |  |  |  |
| Asian | 62 (1.1) | 36 (2.2) | 20 (2.1) |  |  |  |  |
| Other | 146 (2.5) | 82 (5.0) | 31 (3.3) |  |  |  |  |
| Blood pressure |  |  |  |  |  |  |  |
| Systolic | 134.6 ± 16.7 | 135.0 ± 16.6 | 134.3 ± 17.4 | 0.5521 | 0.99 | 0.99 | 0.918 |
| Diastolic | 77.7 ± 10.3 | 76.4 ± 10.6 | 72.9 ± 11.1 | <0.0001 | <0.001 | <0.001 | <0.001 |
| Heart rate -beats/min | 70.5 ± 11.4 | 71.6 ± 11.0 | 72.1 ± 11.4 | <0.0001 | 0.002 | <0.001 | 0.745 |
| BMI | 29.4 ± 5.8 | 31.9 ± 6.7 | 34.0 ± 7.5 | <0.0001 | <0.001 | <0.001 | <0.001 |
| NYHA class III-IV, n (%) | 3262 (56.1) | 989 (60.2) | 577 (61.1) | 0.0007 | 0.003 | 0.004 | 0.642 |
| LVEF -% | 58.2 ± 8.9 | 58.1 ± 8.8 | 58.1 ± 9.0 | 0.9578 | 0.99 | 0.99 | 0.99 |
| Etiology, n (%) |  |  |  | <0.0001 | 0.071 | <0.001 | <0.001 |
| Ischemic | 1688 (34.9) | 458 (36.7) | 259 (44.1) |  |  |  |  |
| Hypertensive | 2393 (49.5) | 627 (50.2) | 230 (39.2) |  |  |  |  |
| Other | 755 (15.6) | 163 (13.1) | 98 (16.7) |  |  |  |  |
| HF duration, n (%) |  |  |  | 0.0019 | 0.001 | 0.071 | 0.369 |
| ≤1 year | 2280 (47.2) | 551 (44.2) | 280 (47.7) |  |  |  |  |
| >1 and ≤5 years | 1800 (37.3) | 448 (35.9) | 197 (33.6) |  |  |  |  |
| >5 years | 752 (15.6) | 248 (19.9) | 110 (18.7) |  |  |  |  |
| Medical history, n (%) |  |  |  |  |  |  |  |
| Current smoking | 321 (11.4) | 77 (9.1) | 42 (6.9) | 0.0021 | 0.06 | 0.001 | 0.141 |
| HF hospitalization within the  past 6 months | 2309 (39.7) | 706 (43.0) | 459 (48.6) | <0.0001 | 0.018 | <0.001 | 0.005 |
| Myocardial infarction | 1550 (26.7) | 519 (31.6) | 293 (31.0) | <0.0001 | <0.001 | 0.005 | 0.771 |
| Angina | 2565 (44.1) | 751 (45.7) | 438 (46.4) | 0.2787 | 0.254 | 0.192 | 0.735 |
| CABG or PCI | 1118 (19.2) | 442 (26.9) | 362 (38.3) | <0.0001 | <0.001 | <0.001 | <0.001 |
| Hypertension | 4566 (78.5) | 1465 (89.2) | 843 (89.3) | <0.0001 | <0.001 | <0.001 | 0.915 |
| Atrial fibrillation | 1914 (32.9) | 520 (31.6) | 269 (28.5) | 0.0228 | 0.33 | 0.007 | 0.094 |
| Stroke | 481 (8.3) | 181 (11.0) | 113 (12.0) | <0.0001 | 0.001 | <0.001 | 0.462 |
| Dyslipidaemia | 1773 (44.6) | 739 (62.1) | 522 (75.0) | <0.0001 | <0.001 | <0.001 | <0.001 |
| Medications, n (%) |  |  |  |  |  |  |  |
| Diuretics | 4609 (79.4) | 1407 (85.7) | 845 (89.6) | <0.0001 | <0.001 | <0.001 | 0.004 |
| Loop | 3243 (55.8) | 1081 (65.8) | 752 (79.7) | <0.0001 | <0.001 | <0.001 | <0.001 |
| Thiazide | 1708 (29.4) | 426 (25.9) | 210 (22.3) | <0.0001 | 0.006 | <0.001 | 0.037 |
| Calcium channel blocker | 2080 (35.8) | 659 (40.1) | 389 (41.3) | 0.0002 | 0.001 | 0.001 | 0.578 |
| ACEI or ARB | 3555 (61.2) | 1163 (70.8) | 713 (75.5) | <0.0001 | <0.001 | <0.001 | 0.009 |
| MRA | 1151 (19.8) | 372 (22.7) | 273 (28.9) | <0.0001 | 0.011 | <0.001 | <0.001 |
| β-blocker | 3524 (60.7) | 1020 (62.1) | 643 (68.2) | <0.0001 | 0.293 | <0.001 | 0.002 |
| Digoxin | 995 (17.1) | 293 (17.8) | 149 (15.8) | 0.4139 | 0.502 | 0.311 | 0.184 |
| Antiarrhythmic agent | 574 (9.9) | 130 (7.9) | 55 (5.8) | <0.0001 | 0.016 | <0.001 | 0.048 |
| Antiplatelet | 3346 (57.6) | 1039 (63.3) | 626 (66.4) | <0.0001 | <0.001 | <0.001 | 0.112 |
| Oral anticoagulant | 1440 (24.8) | 383 (23.3) | 219 (23.2) | 0.3301 | 0.22 | 0.298 | 0.953 |
| Lipid lowering agent | 2062 (35.5) | 818 (49.8) | 608 (64.5) | <0.0001 | <0.001 | <0.001 | <0.001 |
| ECG findings, n (%) |  |  |  |  |  |  |  |
| Atrial fibrillation/flutter | 1113 (19.2) | 303 (18.5) | 141 (15.0) | 0.009 | 0.489 | 0.002 | 0.026 |
| QRS duration | 90 (80-108) | 94 (80-110) | 94 (82-113) | 0.0004 | 0.0313 | 0.0004 | 0.1938 |
| Bundle branch block | 857 (14.8) | 270 (16.4) | 163 (17.4) | 0.0558 | 0.1 | 0.042 | 0.549 |
| Left bundle branch block | 247 (8.2) | 59 (7.4) | 30 (8.8) | 0.6618 | 0.446 | 0.705 | 0.413 |
| Right bundle branch block | 185 (6.2) | 70 (8.8) | 28 (8.3) | 0.019 | 0.009 | 0.138 | 0.765 |
| Left ventricular hypertrophy | 1311 (22.6) | 341 (20.8) | 146 (15.5) | <0.0001 | 0.109 | <0.001 | 0.001 |
| Symptoms and signs, n (%) |  |  |  |  |  |  |  |
| Dyspnea on exertion | 2754 (97.7) | 827 (97.5) | 590 (97.5) | 0.9405 | 0.773 | 0.798 | 0.997 |
| Orthopnea | 572 (20.4) | 234 (27.8) | 228 (37.9) | <0.0001 | <0.001 | <0.001 | <0.001 |
| Paroxsymal noctural dyspnea | 320 (11.4) | 123 (14.7) | 110 (18.4) | <0.0001 | 0.011 | <0.001 | 0.058 |
| Dyspnea at rest | 142 (7.7) | 55 (12.1) | 38 (15.3) | <0.0001 | 0.003 | <0.001 | 0.235 |
| Jugular venous distention | 533 (9.3) | 177 (10.9) | 118 (12.8) | 0.0014 | 0.044 | 0.001 | 0.157 |
| Edema | 2759 (47.5) | 892 (54.3) | 604 (64.0) | <0.0001 | <0.001 | <0.001 | <0.001 |
| Rales | 1258 (21.7) | 362 (22.2) | 229 (24.5) | 0.1632 | 0.7 | 0.057 | 0.176 |
| Third heart sound | 299 (6.2) | 93 (7.5) | 52 (8.9) | 0.0229 | 0.105 | 0.013 | 0.297 |
| Hepatomegaly | 647 (13.4) | 205 (16.5) | 61 (10.5) | 0.0012 | 0.006 | 0.047 | 0.001 |
| Health-related quality of life |  |  |  |  |  |  |  |
| Minnesota Living With Heart  Failure score | 40 (26-56) | 45 (27-62) | 51 (32-69) | <0.0001 | <0.0001 | <0.0001 | 0.0017 |
| KCCQ clinical summary score | 65 (46-80) | 58 (41-75) | 52 (33-69) | <0.0001 | 0.0002 | <0.0001 | 0.0013 |
| Laboratory measurements |  |  |  |  |  |  |  |
| eGFR - ml/min/1.73m^2^ | 71.5 ± 22.2 | 71.4 ± 24.8 | 62.5 ± 23.3 | <0.0001 | 0.99 | <0.001 | <0.001 |
| eGFR <60 ml/min/1.73m^2^, n (%) | 1451 (32.4) | 511 (36.9) | 439 (53.0) | <0.0001 | 0.002 | <0.001 | <0.001 |
| Haemoglobin - g/dL | 13.9 ± 1.6 | 13.5 ± 1.7 | 12.9 ± 1.7 | <0.0001 | <0.001 | <0.001 | <0.001 |
| NT-proBNP - pg/mL | 364  (139-1017) | 429  (165-1041) | 564  (207-1334) | <0.0001 | 0.1902 | <0.0001 | 0.003 |

Plus-minus values are mean ± standard deviation. Duration of diabetes, QRS duration, Minnesota Living with Heart Failure score, KCCQ clinical summary score and NT-proBNP are presented as median with interquartile range.

LVEF denotes left ventricular ejection fraction; NYHA, New York Heart Association; CABG, coronary artery bypass grafting; PCI, percutaneous coronary intervention; ACEI, angiotensin converting enzyme inhibitor; ARB, angiotensin receptor blocker; MRA, mineralocorticoid receptor antagonist; ECG, electrocardiogram; HF, heart failure; DM, diabetes mellitus; KCCQ, the Kansas City Cardiomyopathy Questionnaire; eGFR, estimated glomerular filtration rate; NT pro-BNP, N terminal pro-B type natriuretic peptide.

Age at diabetes onset and duration of diabetes were available in 686 (98%) patients from CHARM-Preserved and 743 (99%) from TOPCAT; NT-proBNP was available in 3832 (46%) patients; eGFR was available in 6689 (80%) patients; hemoglobin was available in 5699 (68%) patients.

Minnesota Living with Heart Failure score was available in 773 (30%) patients from CHARM-Preserved and 3148 (76%) patients from I-Preserve, and possible scores range from 0 to 105, with lower scores indicating a better quality of life.

KCCQ clinical summary score was available in 1690 (98%) patients from TOPCAT, and possible scores range from 0 to 100, with higher scores indicating better health-related quality of life.

Table S2. Clinical outcomes according to baseline diabetes mellitus status and insulin use in the combined data sets of CHARM-Preserved (LVEF ≥45%), I-Preserve and TOPCAT (Americas) **after excluding patients with age at diabetes onset below 20 years**

|  | Patients, n | Events, n (%) | Annual rate, per 100 person-years (95% CI) | Unadjusted HR† (95% CI) | Adjusted1HR† (95% CI) | Adjusted2 HR† (95% CI) |
| --- | --- | --- | --- | --- | --- | --- |
| CV death or HF hospitalization |  |  |  |  |  |  |
| No DM | 5813 | 1227 (21.1) | 6.3 (6.0-6.7) | 1.00 (Referent) | 1.00 (Referent) | 1.00 (Referent) |
| DM not on insulin | 1643 | 507 (30.9) | 10.3 (9.4-11.2) | 1.59 (1.43-1.76), p<0.001 | 1.54 (1.39-1.72), p<0.001 | 1.51 (1.35-1.68), p<0.001 |
| DM on insulin | 944 | 400 (42.4) | 16.9 (15.3-18.7) | 2.45 (2.18-2.75), p<0.001 | 2.35 (2.08-2.65), p<0.001 | 2.19 (1.94-2.48), p<0.001 |
| HF hospitalization |  |  |  |  |  |  |
| No DM | 5813 | 811 (14.0) | 4.2 (3.9-4.5) | 1.00 (Referent) | 1.00 (Referent) | 1.00 (Referent) |
| DM not on insulin | 1643 | 351 (21.4) | 7.1 (6.4-7.9) | 1.63 (1.44-1.85), p<0.001 | 1.57 (1.38-1.78), p<0.001 | 1.52 (1.34-1.73), p<0.001 |
| DM on insulin | 944 | 313 (33.2) | 13.2 (11.9-14.8) | 2.76 (2.41-3.15), p<0.001 | 2.48 (2.15-2.86), p<0.001 | 2.27 (1.96-2.62), p<0.001 |
| CV death |  |  |  |  |  |  |
| No DM | 5813 | 678 (11.7) | 3.2 (3.0-3.5) | 1.00 (Referent) | 1.00 (Referent) | 1.00 (Referent) |
| DM not on insulin | 1643 | 254 (15.5) | 4.6 (4.0-5.2) | 1.41 (1.22-1.63), p<0.001 | 1.45 (1.25-1.68), p<0.001 | 1.42 (1.23-1.65), p<0.001 |
| DM on insulin | 944 | 174 (18.4) | 5.9 (5.1-6.9) | 1.84 (1.56-2.19), p<0.001 | 2.08 (1.74-2.49), p<0.001 | 1.94 (1.62-2.32), p<0.001 |
| All-cause death |  |  |  |  |  |  |
| No DM | 5813 | 1024 (17.6) | 4.9 (4.6-5.2) | 1.00 (Referent) | 1.00 (Referent) | 1.00 (Referent) |
| DM not on insulin | 1643 | 381 (23.2) | 6.8 (6.2-7.6) | 1.39 (1.23-1.56), p<0.001 | 1.44 (1.28-1.63), p<0.001 | 1.42 (1.25-1.60), p<0.001 |
| DM on insulin | 944 | 259 (27.4) | 8.8 (7.8-10.0) | 1.75 (1.52-2.01), p<0.001 | 2.01 (1.74-2.33), p<0.001 | 1.86 (1.61-2.16), p<0.001 |
| Sudden death |  |  |  |  |  |  |
| No DM | 5813 | 235 (4.0) | 1.1 (1.0-1.3) | 1.00 (Referent) | 1.00 (Referent) | 1.00 (Referent) |
| DM not on insulin | 1643 | 91 (5.5) | 1.6 (1.3-2.0) | 1.48 (1.16-1.89), p=0.001 | 1.46 (1.14-1.88), p=0.003 | 1.46 (1.14-1.87), p=0.003 |
| DM on insulin | 944 | 74 (7.8) | 2.5 (2.0-3.2) | 2.40 (1.84-3.13), p<0.001 | 2.69 (2.03-3.56), p<0.001 | 2.55 (1.92-3.38), p<0.001 |
| Pump failure death |  |  |  |  |  |  |
| No DM | 5813 | 158 (2.7) | 0.8 (0.6-0.9) | 1.00 (Referent) | 1.00 (Referent) | 1.00 (Referent) |
| DM not on insulin | 1643 | 68 (4.1) | 1.2 (1.0-1.5) | 1.61 (1.21-2.14), p=0.001 | 1.92 (1.43-2.58), p<0.001 | 1.84 (1.37-2.48), p<0.001 |
| DM on insulin | 944 | 40 (4.2) | 1.4 (1.0-1.9) | 1.74 (1.22-2.47), p=0.002 | 2.37 (1.63-3.44), p<0.001 | 2.13 (1.46-3.11), p<0.001 |

CV indicates cardiovascular; DM, diabetes mellitus; HF, heart failure.

Adjustment Model 1: age, sex, heart rate, diastolic blood pressure, LVEF, NYHA class III/IV, BMI, HF hospitalisation with the past 6 months, history of myocardial infarction, hypertension, and atrial fibrillation.

Adjustment Model 2: age, sex, heart rate, diastolic blood pressure, LVEF, NYHA class III/IV, BMI, HF hospitalisation with the past 6 months, history of myocardial infarction, hypertension, and atrial fibrillation, eGFR, and log NT-proBNP with simple imputation of eGFR and NT-proBNP.

†Hazard ratios for combined data were adjusted for within-trial clustering.

Table S3. Clinical outcomes according to baseline insulin use in diabetic patients in the combined data sets of CHARM-Preserved (LVEF ≥45%), I-Preserve and TOPCAT (Americas) **after excluding patients with age at diabetes onset below 20 years**

|  | Unadjusted HR† (95% CI) | Adjusted1 HR† (95% CI) | Adjusted2 HR† (95% CI) | Adjusted3 HR† (95% CI) |
| --- | --- | --- | --- | --- |
| CV death or HF hospitalization |  |  |  |  |
| DM not on insulin | 1.00 (Referent) | 1.00 (Referent) | 1.00 (Referent) | 1.00 (Referent) |
| DM on insulin | 1.55 (1.36-1.77), p<0.001 | 1.48 (1.29-1.70), p<0.001 | 1.41 (1.23-1.62), p<0.001 | 1.38 (1.19-1.60), p<0.001 |
| HF hospitalization |  |  |  |  |
| DM not on insulin | 1.00 (Referent) | 1.00 (Referent) | 1.00 (Referent) | 1.00 (Referent) |
| DM on insulin | 1.69 (1.45-1.97), p<0.001 | 1.55 (1.32-1.82), p<0.001 | 1.43 (1.22-1.68), p<0.001 | 1.40 (1.18-1.67), p<0.001 |
| CV death |  |  |  |  |
| DM not on insulin | 1.00 (Referent) | 1.00 (Referent) | 1.00 (Referent) | 1.00 (Referent) |
| DM on insulin | 1.33 (1.10-1.62), p=0.004 | 1.42 (1.16-1.73), p=0.001 | 1.34 (1.10-1.65), p=0.004 | 1.31 (1.05-1.61), p=0.014 |
| All-cause death |  |  |  |  |
| DM not on insulin | 1.00 (Referent) | 1.00 (Referent) | 1.00 (Referent) | 1.00 (Referent) |
| DM on insulin | 1.29 (1.10-1.52), p=0.002 | 1.37 (1.16-1.62), p<0.001 | 1.29 (1.09-1.53), p=0.003 | 1.24 (1.04-1.48), p=0.016 |
| Sudden death |  |  |  |  |
| DM not on insulin | 1.00 (Referent) | 1.00 (Referent) | 1.00 (Referent) | 1.00 (Referent) |
| DM on insulin | 1.62 (1.18-2.20), p=0.002 | 1.83 (1.33-2.52), p<0.001 | 1.77 (1.28-2.45), p=0.001 | 1.65 (1.18-2.31), p=0.004 |
| Pump failure death |  |  |  |  |
| DM not on insulin | 1.00 (Referent) | 1.00 (Referent) | 1.00 (Referent) | 1.00 (Referent) |
| DM on insulin | 1.14 (0.77-1.69), p=0.518 | 1.27 (0.84-1.91), p=0.250 | 1.16 (0.76-1.75), p=0.489 | 1.18 (0.76-1.81), p=0.464 |

CV indicates cardiovascular; DM, diabetes mellitus; HF, heart failure.

Adjustment Model 1: age, sex, heart rate, diastolic blood pressure, LVEF, NYHA class III/IV, BMI, HF hospitalisation with the past 6 months, history of myocardial infarction, hypertension, and atrial fibrillation.

Adjustment Model 2: age, sex, heart rate, diastolic blood pressure, LVEF, NYHA class III/IV, BMI, HF hospitalisation with the past 6 months, history of myocardial infarction, hypertension, and atrial fibrillation, eGFR, and log NT-proBNP with simple imputation of eGFR and NT-proBNP.

Adjustment Model 3: age, sex, heart rate, diastolic blood pressure, LVEF, NYHA class III/IV, BMI, HF hospitalisation with the past 6 months, history of myocardial infarction, hypertension, and atrial fibrillation, eGFR, log NT-proBNP and log diabetes duration with simple imputation of eGFR, NT-proBNP and diabetes duration.

†Hazard ratios for combined data were adjusted for within-trial clustering.

Table S4. Clinical outcomes according to baseline diabetes mellitus status and insulin use in the combined data sets of CHARM-Preserved (LVEF ≥45%), I-Preserve and TOPCAT (Americas) **in patients with NT-proBNP available** **after excluding patients with age at diabetes onset below 20 years**

|  | Patients, n | Events, n (%) | Annual rate, per 100 person-years (95% CI) | Unadjusted HR† (95% CI) | Adjusted1 HR† (95% CI) | Adjusted2 HR† (95% CI) |
| --- | --- | --- | --- | --- | --- | --- |
| CV death or HF hospitalization |  |  |  |  |  |  |
| No DM | 2728 | 604 (22.1) | 5.9 (5.5-6.4) | 1.00 (Referent) | 1.00 (Referent) | 1.00 (Referent) |
| DM not on insulin | 767 | 232 (30.3) | 8.9 (7.8-10.1) | 1.48 (1.27-1.72), p<0.001 | 1.44 (1.23-1.68), p<0.001 | 1.42 (1.21-1.66), p<0.001 |
| DM on insulin | 337 | 134 (39.8) | 13.9 (11.7-16.4) | 2.25 (1.86-2.71), p<0.001 | 2.27 (1.86-2.75), p<0.001 | 2.07 (1.70-2.52), p<0.001 |
| HF hospitalization |  |  |  |  |  |  |
| No DM | 2728 | 368 (13.5) | 3.6 (3.3-4.0) | 1.00 (Referent) | 1.00 (Referent) | 1.00 (Referent) |
| DM not on insulin | 767 | 149 (19.4) | 5.7 (4.9-6.7) | 1.54 (1.27-1.86), p<0.001 | 1.48 (1.22-1.80), p<0.001 | 1.45 (1.20-1.77), p<0.001 |
| DM on insulin | 337 | 91 (27.0) | 9.4 (7.7-11.6) | 2.45 (1.94-3.08), p<0.001 | 2.34 (1.84-2.97), p<0.001 | 2.09 (1.64-2.66), p<0.001 |
| CV death |  |  |  |  |  |  |
| No DM | 2728 | 358 (13.1) | 3.3 (3.0-3.6) | 1.00 (Referent) | 1.00 (Referent) | 1.00 (Referent) |
| DM not on insulin | 767 | 130 (17.0) | 4.5 (3.8-5.3) | 1.36 (1.12-1.67), p=0.002 | 1.36 (1.10-1.67), p=0.004 | 1.35 (1.09-1.65), p=0.005 |
| DM on insulin | 337 | 73 (21.7) | 6.3 (5.0-7.9) | 1.91 (1.48-2.46), p<0.001 | 2.03 (1.56-2.63), p<0.001 | 1.80 (1.38-2.35), p<0.001 |
| All-cause death |  |  |  |  |  |  |
| No DM | 2728 | 518 (19.0) | 4.7 (4.3-5.2) | 1.00 (Referent) | 1.00 (Referent) | 1.00 (Referent) |
| DM not on insulin | 767 | 188 (24.5) | 6.5 (5.6-7.5) | 1.36 (1.15-1.61), p<0.001 | 1.36 (1.14-1.61), p<0.001 | 1.35 (1.14-1.61), p=0.001 |
| DM on insulin | 337 | 103 (30.6) | 8.9 (7.3-10.8) | 1.86 (1.50-2.30), p<0.001 | 2.05 (1.64-2.55), p<0.001 | 1.82 (1.46-2.27), p<0.001 |
| Sudden death |  |  |  |  |  |  |
| No DM | 2728 | 129 (4.7) | 1.2 (1.0-1.4) | 1.00 (Referent) | 1.00 (Referent) | 1.00 (Referent) |
| DM not on insulin | 767 | 49 (6.4) | 1.7 (1.3-2.2) | 1.43 (1.03-1.99), p=0.033 | 1.42 (1.01-1.99), p=0.042 | 1.41 (1.01-1.98), p=0.045 |
| DM on insulin | 337 | 35 (10.4) | 3.0 (2.2-4.2) | 2.56 (1.76-3.72), p<0.001 | 2.86 (1.93-4.24), p<0.001 | 2.57 (1.73-3.83), p<0.001 |
| Pump failure death |  |  |  |  |  |  |
| No DM | 2728 | 68 (2.5) | 0.6 (0.5-0.8) | 1.00 (Referent) | 1.00 (Referent) | 1.00 (Referent) |
| DM not on insulin | 767 | 30 (3.9) | 1.0 (0.7-1.5) | 1.63 (1.06-2.50), p=0.027 | 1.77 (1.14-2.76), p=0.011 | 1.77 (1.14-2.77), p=0.012 |
| DM on insulin | 337 | 16 (4.8) | 1.4 (0.8-2.2) | 2.14 (1.24-3.71), p=0.006 | 2.46 (1.40-4.32), p=0.002 | 2.19 (1.24-3.87), p=0.007 |

Data were available in 3832 patients.

CV indicates cardiovascular; DM, diabetes mellitus; HF, heart failure.

Adjustment Model 1: age, sex, heart rate, diastolic blood pressure, LVEF, NYHA class III/IV, BMI, HF hospitalisation with the past 6 months, history of myocardial infarction, hypertension, and atrial fibrillation.

Adjustment Model 2: age, sex, heart rate, diastolic blood pressure, LVEF, NYHA class III/IV, BMI, HF hospitalisation with the past 6 months, history of myocardial infarction, hypertension, and atrial fibrillation, eGFR, and log NT-proBNP.

†Hazard ratios for combined data were adjusted for within-trial clustering.

Table S5. Clinical outcomes according to baseline insulin use in diabetic patients in the combined data sets of CHARM-Preserved (LVEF ≥45%), I-Preserve and TOPCAT (Americas) **in patients with NT-proBNP available** **after excluding patients with age at diabetes onset below 20 years**

|  | Unadjusted HR† (95% CI) | Adjusted1 HR† (95% CI) | Adjusted2 HR† (95% CI) | Adjusted3 HR† (95% CI) |
| --- | --- | --- | --- | --- |
| CV death or HF hospitalization |  |  |  |  |
| DM not on insulin | 1.00 (Referent) | 1.00 (Referent) | 1.00 (Referent) | 1.00 (Referent) |
| DM on insulin | 1.52 (1.22-1.88), p<0.001 | 1.52 (1.22-1.89), p<0.001 | 1.41 (1.13-1.76), p=0.002 | 1.38 (1.11-1.73), p=0.004 |
| HF hospitalization |  |  |  |  |
| DM not on insulin | 1.00 (Referent) | 1.00 (Referent) | 1.00 (Referent) | 1.00 (Referent) |
| DM on insulin | 1.58 (1.22-2.05), p=0.001 | 1.53 (1.17-2.00), p=0.002 | 1.35 (1.02-1.77), p=0.033 | 1.34 (1.01-1.76), p=0.04 |
| CV death |  |  |  |  |
| DM not on insulin | 1.00 (Referent) | 1.00 (Referent) | 1.00 (Referent) | 1.00 (Referent) |
| DM on insulin | 1.40 (1.05-1.87), p=0.021 | 1.44 (1.07-1.93), p=0.016 | 1.33 (0.99-1.80), p=0.059 | 1.30 (0.96-1.75), p=0.088 |
| All-cause death |  |  |  |  |
| DM not on insulin | 1.00 (Referent) | 1.00 (Referent) | 1.00 (Referent) | 1.00 (Referent) |
| DM on insulin | 1.38 (1.08-1.75), p=0.009 | 1.46 (1.14-1.87), p=0.003 | 1.32 (1.03-1.70), p=0.028 | 1.29 (1.00-1.66), p=0.047 |
| Sudden death |  |  |  |  |
| DM not on insulin | 1.00 (Referent) | 1.00 (Referent) | 1.00 (Referent) | 1.00 (Referent) |
| DM on insulin | 1.77 (1.15-2.74), p=0.01 | 1.92 (1.23-3.02), p=0.004 | 1.87 (1.18-2.95), p=0.007 | 1.79 (1.13-2.82), p=0.013 |
| Pump failure death |  |  |  |  |
| DM not on insulin | 1.00 (Referent) | 1.00 (Referent) | 1.00 (Referent) | 1.00 (Referent) |
| DM on insulin | 1.34 (0.73-2.46), p=0.344 | 1.27 (0.67-2.38), p=0.461 | 1.12 (0.60-2.12), p=0.718 | 1.15 (0.61-2.18), p=0.664 |

CV indicates cardiovascular; DM, diabetes mellitus; HF, heart failure.

Adjustment Model 1: age, sex, heart rate, diastolic blood pressure, LVEF, NYHA class III/IV, BMI, HF hospitalisation with the past 6 months, history of myocardial infarction, hypertension, and atrial fibrillation.

Adjustment Model 2: age, sex, heart rate, diastolic blood pressure, LVEF, NYHA class III/IV, BMI, HF hospitalisation with the past 6 months, history of myocardial infarction, hypertension, and atrial fibrillation, eGFR, and log NT-proBNP.

Adjustment Model 3: age, sex, heart rate, diastolic blood pressure, LVEF, NYHA class III/IV, BMI, HF hospitalisation with the past 6 months, history of myocardial infarction, hypertension, and atrial fibrillation, eGFR, log NT-proBNP and log diabetes duration, with simple imputation of diabetes duration.

†Hazard ratios for combined data were adjusted for within-trial clustering.

Table S6. Baseline characteristics according to baseline diabetes mellitus status and insulin use among patients with full echocardiographic examination

|  | No DM | DM not on insulin | DM on insulin | p value |
| --- | --- | --- | --- | --- |
|  | n=903 | n=289 | n=206 | overall |
| Age -years | 72.1 ± 8.3 | 71.4 ± 8.4 | 68.8 ± 8.6 | <0.0001 |
| Age of diabetes onset -years |  | 59.2 ± 13.3 | 49.4 ± 14.9 |  |
| Duration of diabetes -years |  | 9 (4-15) | 16 (10-22) |  |
| Male sex, n (%) | 370 (41.0) | 156 (54.0) | 98 (47.6) | 0.0004 |
| Race, n (%) |  |  |  | <0.0001 |
| White | 802 (88.8) | 245 (84.8) | 154 (74.8) |  |
| Black | 67 (7.4) | 31 (10.7) | 41 (19.9) |  |
| Asian | 3 (0.3) | 0 (0.0) | 1 (0.5) |  |
| Other | 31 (3.4) | 13 (4.5) | 10 (4.9) |  |
| Blood pressure |  |  |  |  |
| Systolic | 132.0 ± 15.4 | 132.6 ± 15.5 | 130.7 ± 18.0 | 0.4009 |
| Diastolic | 76.1 ± 10.4 | 75.6 ± 10.6 | 70.0 ± 10.8 | <0.0001 |
| Heart rate -beats/min | 69.0 ± 11.0 | 70.0 ± 10.8 | 71.1 ± 11.7 | 0.0318 |
| Body mass index | 30.3 ± 6.4 | 32.9 ± 6.8 | 36.0 ± 7.7 | <0.0001 |
| NYHA class III-IV, n (%) | 538 (59.6) | 172 (59.5) | 107 (51.9) | 0.1223 |
| LVEF -% | 59.3 ± 8.2 | 58.9 ± 8.7 | 58.7 ± 8.0 | 0.5537 |
| Etiology, n (%) |  |  |  | 0.2228 |
| Ischemic | 105 (18.8) | 32 (22.9) | 13 (27.7) |  |
| Hypertensive | 408 (73.1) | 93 (66.4) | 28 (59.6) |  |
| Other | 45 (8.1) | 15 (10.7) | 6 (12.8) |  |
| HF duration, n (%) |  |  |  | 0.0196 |
| ≤1 year | 232 (41.6) | 49 (35.0) | 26 (55.3) |  |
| >1 and ≤5 years | 240 (43.0) | 59 (42.1) | 11 (23.4) |  |
| >5 years | 86 (15.4) | 32 (22.9) | 10 (21.3) |  |
| Medical history, n (%) |  |  |  |  |
| Current smoking | 26 (7.5) | 5 (3.4) | 10 (6.3) | 0.2133 |
| HF hospitalization within the past  6 months | 346 (38.3) | 134 (46.4) | 107 (51.9) | 0.0004 |
| Myocardial infarction | 164 (18.2) | 75 (26.0) | 47 (22.8) | 0.0112 |
| Angina | 311 (34.4) | 97 (33.6) | 65 (31.6) | 0.7274 |
| CABG or PCI | 146 (16.2) | 76 (26.3) | 78 (37.9) | <0.0001 |
| Hypertension | 808 (89.5) | 276 (95.5) | 194 (94.2) | 0.002 |
| Atrial fibrillation | 309 (34.2) | 95 (32.9) | 69 (33.5) | 0.9094 |
| Stroke | 75 (8.3) | 30 (10.4) | 25 (12.1) | 0.1806 |
| Dyslipidaemia | 422 (46.7) | 191 (66.1) | 159 (77.2) | <0.0001 |
| Medications, n (%) |  |  |  |  |
| Diuretics | 756 (83.7) | 250 (86.5) | 194 (94.2) | 0.0005 |
| Loop | 521 (57.7) | 203 (70.2) | 176 (85.4) | 0.0001 |
| Thiazide | 337 (37.3) | 83 (28.7) | 57 (27.7) | 0.0029 |
| Calcium channel blocker | 364 (40.3) | 133 (46.0) | 86 (41.7) | 0.2303 |
| ACEI or ARB | 612 (67.8) | 222 (76.8) | 172 (83.5) | <0.0001 |
| MRA | 264 (29.2) | 97 (33.6) | 91 (44.2) | 0.0002 |
| β-blocker | 624 (69.1) | 204 (70.6) | 169 (82.0) | 0.001 |
| Digoxin | 83 (9.2) | 32 (11.1) | 15 (7.3) | 0.3527 |
| Antiarrhythmic agent | 80 (8.9) | 21 (7.3) | 9 (4.4) | 0.0886 |
| Antiplatelet | 497 (55.0) | 180 (62.3) | 146 (70.9) | <0.0001 |
| Oral anticoagulant | 237 (26.2) | 73 (25.3) | 53 (25.7) | 0.9428 |
| Lipid lowering agent | 350 (38.8) | 166 (57.4) | 160 (77.7) | <0.0001 |
| ECG findings, n (%) |  |  |  |  |
| Atrial fibrillation/flutter | 177 (19.7) | 60 (20.8) | 37 (18.0) | 0.7562 |
| QRS duration | 90 (80-108) | 96 (84-114) | 94 (85-110) | 0.0014 |
| Bundle branch block | 138 (15.3) | 49 (17.0) | 36 (17.6) | 0.6487 |
| Left bundle branch block | 37 (6.6) | 7 (5.0) | 4 (8.5) | 0.6539 |
| Right bundle branch block | 34 (6.1) | 10 (7.1) | 3 (6.4) | 0.9007 |
| Left ventricular hypertrophy | 199 (22.1) | 56 (19.4) | 21 (10.2) | 0.0006 |
| Symptoms and signs, n (%) |  |  |  |  |
| Dyspnea on exertion | 339 (98.3) | 148 (99.3) | 156 (98.1) | 0.6178 |
| Orthopnea | 106 (31.2) | 47 (32.2) | 72 (45.6) | 0.0054 |
| Paroxsymal noctural dyspnea | 51 (15.0) | 22 (15.2) | 26 (16.8) | 0.8742 |
| Dyspnea at rest |  |  |  |  |
| Jugular venous distention | 124 (14.0) | 40 (14.2) | 39 (19.9) | 0.1061 |
| Edema | 564 (62.5) | 198 (68.5) | 152 (73.8) | 0.0042 |
| Rales | 196 (21.9) | 55 (19.2) | 41 (20.2) | 0.5935 |
| Third heart sound | 32 (5.7) | 4 (2.9) | 5 (10.6) | 0.115 |
| Hepatomegaly | 105 (18.8) | 30 (21.4) | 5 (10.6) | 0.2611 |
| Health-related quality of life |  |  |  |  |
| Minnesota Living With Heart Failure score | 42 (28-55) | 43 (26-60) | 45 (30-58) | 0.7181 |
| KCCQ clinical summary score | 60 (44-80) | 58 (38-75) | 52 (32-69) | <0.0001 |
| Laboratory measurements |  |  |  |  |
| eGFR - ml/min/1.73m^2^ | 70.2 ± 22.2 | 69.7 ± 24.5 | 58.7 ± 19.6 | <0.0001 |
| eGFR <60 ml/min/1.73m^2^, n (%) | 315 (35.4) | 107 (37.7) | 125 (61.6) | <0.0001 |
| Hemoglobin - g/dL | 13.7 ± 1.5 | 13.2 ± 1.7 | 12.4 ± 1.6 | <0.0001 |
| NT-proBNP - pg/mL | 341 (131-921) | 572 (222-1303) | 704 (310-1820) | <0.0001 |

Echocardiographic data was available in 745 patients in I-Preserve and 653 patients in TOPCAT (Americas).

Plus-minus values are mean ± standard deviation. Duration of diabetes, QRS duration, Minnesota Living with Heart Failure score, KCCQ clinical summary score and NT-proBNP are presented as median with interquartile range.

LVEF denotes left ventricular ejection fraction; NYHA, New York Heart Association; CABG, coronary artery bypass grafting; PCI, percutaneous coronary intervention; ACEI, angiotensin converting enzyme inhibitor; ARB, angiotensin receptor blocker; MRA, mineralocorticoid receptor antagonist; ECG, electrocardiogram; HF, heart failure; DM, diabetes mellitus; KCCQ, the Kansas City Cardiomyopathy Questionnaire; eGFR, estimated glomerular filtration rate; NT pro-BNP, N terminal pro-B type natriuretic peptide.

Age at diabetes onset and duration of diabetes were available in 302 (98%) from TOPCAT; NT-proBNP was available in 790 (57%) patients; eGFR was available in 1378 (99%) patients; hemoglobin was available in 1358 (97%) patients.

Minnesota Living with Heart Failure score was available in 561 (75%) patients from I-Preserve, and possible scores range from 0 to 105, with lower scores indicating a better quality of life.

KCCQ clinical summary score was available in 630 (96%) patients from TOPCAT, and possible scores range from 0 to 100, with higher scores indicating better health-related quality of life.

Table S7. Clinical outcomes according to baseline diabetes mellitus status and insulin use in the combined data sets of CHARM-Preserved (LVEF ≥45%), I-Preserve and TOPCAT (Americas) (Only Patients with Echocardiographic Data)

|  | patients, n | Event, n (%) | Annual rate, per 100 person-years (95% CI) | Unadjusted HR† (95% CI) | Adjusted1 HR† (95% CI) | Adjusted2 HR† (95% CI) |
| --- | --- | --- | --- | --- | --- | --- |
| CV death or HF hospitalization |  |  |  |  |  |  |
| No DM | 903 | 187 (20.7) | 6.2 (5.4-7.2) | 1.00 (Referent) | 1.00 (Referent) | 1.00 (Referent) |
| DM not on insulin | 289 | 86 (29.8) | 9.9 (8.0-12.2) | 1.45 (1.12-1.88), p=0.004 | 1.13 (0.86-1.48), p=0.373 | 1.09 (0.83-1.43), p=0.536 |
| DM on insulin | 206 | 82 (39.8) | 16.2 (13-20.1) | 2.05 (1.56-2.70), p<0.001 | 1.66 (1.23-2.25), p=0.001 | 1.60 (1.18-2.17), p=0.002 |
| HF hospitalization |  |  |  |  |  |  |
| No DM | 903 | 127 (14.1) | 4.2 (3.6-5.0) | 1.00 (Referent) | 1.00 (Referent) | 1.00 (Referent) |
| DM not on insulin | 289 | 62 (21.5) | 7.1 (5.6-9.1) | 1.51 (1.11-2.05), p=0.009 | 1.19 (0.86-1.64), p=0.286 | 1.13 (0.82-1.56), p=0.444 |
| DM on insulin | 206 | 65 (31.6) | 12.8 (10.0-16.3) | 2.27 (1.66-3.10), p<0.001 | 1.75 (1.23-2.48), p=0.002 | 1.67 (1.17-2.37), p=0.004 |
| CV death |  |  |  |  |  |  |
| No DM | 903 | 84 (9.3) | 2.6 (2.1-3.2) | 1.00 (Referent) | 1.00 (Referent) | 1.00 (Referent) |
| DM not on insulin | 289 | 39 (13.5) | 4.0 (2.9-5.4) | 1.49 (1.01-2.18), p=0.042 | 1.32 (0.89-1.98), p=0.171 | 1.29 (0.86-1.93), p=0.216 |
| DM on insulin | 206 | 29 (14.1) | 4.7 (3.2-6.7) | 1.63 (1.05-2.53), p=0.03 | 1.64 (1.01-2.67), p=0.044 | 1.58 (0.97-2.57), p=0.066 |
| All-cause death |  |  |  |  |  |  |
| No DM | 903 | 143 (15.8) | 4.4 (3.7-5.2) | 1.00 (Referent) | 1.00 (Referent) | 1.00 (Referent) |
| DM not on insulin | 289 | 61 (21.1) | 6.2 (4.8-8.0) | 1.36 (1.00-1.83), p=0.048 | 1.23 (0.89-1.68), p=0.205 | 1.19 (0.87-1.64), p=0.283 |
| DM on insulin | 206 | 48 (23.3) | 7.7 (5.8-10.2) | 1.56 (1.11-2.19), p=0.011 | 1.51 (1.04-2.20), p=0.031 | 1.46 (1.00-2.12), p=0.052 |
| Sudden death |  |  |  |  |  |  |
| No DM | 903 | 24 (2.7) | 0.7 (0.5-1.1) | 1.00 (Referent) | 1.00 (Referent) | 1.00 (Referent) |
| DM not on insulin | 289 | 14 (4.8) | 1.4 (0.8-2.4) | 2.02 (1.04-3.92), p=0.038 | 1.56 (0.77-3.14), p=0.214 | 1.61 (0.79-3.25), p=0.186 |
| DM on insulin | 206 | 13 (6.3) | 2.1 (1.2-3.6) | 3.31 (1.62-6.75), p=0.001 | 3.03 (1.37-6.67), p=0.006 | 3.05 (1.37-6.80), p=0.006 |
| Pump failure death |  |  |  |  |  |  |
| No DM | 903 | 16 (1.8) | 0.5 (0.3-0.8) | 1.00 (Referent) | 1.00 (Referent) | 1.00 (Referent) |
| DM not on insulin | 289 | 11 (3.8) | 1.1 (0.6-2.0) | 2.08 (0.96-4.51), p=0.063 | 2.84 (1.24-6.54), p=0.014 | 2.65 (1.14-6.16), p=0.023 |
| DM on insulin | 206 | 4 (1.9) | 0.6 (0.2-1.7) | 1.00 (0.33-3.08), p=0.99 | 1.49 (0.44-4.99), p=0.519 | 1.39 (0.40-4.79), p=0.601 |

Adjustment Model 1: age, sex, black race, heart rate, diastolic blood pressure, LVEF, NYHA class III/IV, BMI, HF hospitalisation with the past 6 months, history of myocardial infarction, hypertension, and atrial fibrillation, eGFR, and log NT-proBNP with simple imputation of eGFR and NT-proBNP.

Adjustment Model 2: age, sex, black race, heart rate, diastolic blood pressure, LVEF, NYHA class III/IV, BMI, HF hospitalisation with the past 6 months, history of myocardial infarction, hypertension, and atrial fibrillation, eGFR, and log NT-proBNP, E/E' septal, LV mass index and LA volume index with simple imputation of eGFR and NT-proBNP, E/E' septal, LV mass index and LA volume index. †Hazard ratios for combined data were adjusted for within-trial clustering.

Figure S1. Cumulative incidences for clinical outcomes according to baseline diabetes mellitus status and insulin use in the combined data sets of CHARM-Preserved (LVEF ≥45%), I-Preserve and TOPCAT (Americas) **after excluding patients with age at diabetes onset below 20 years**


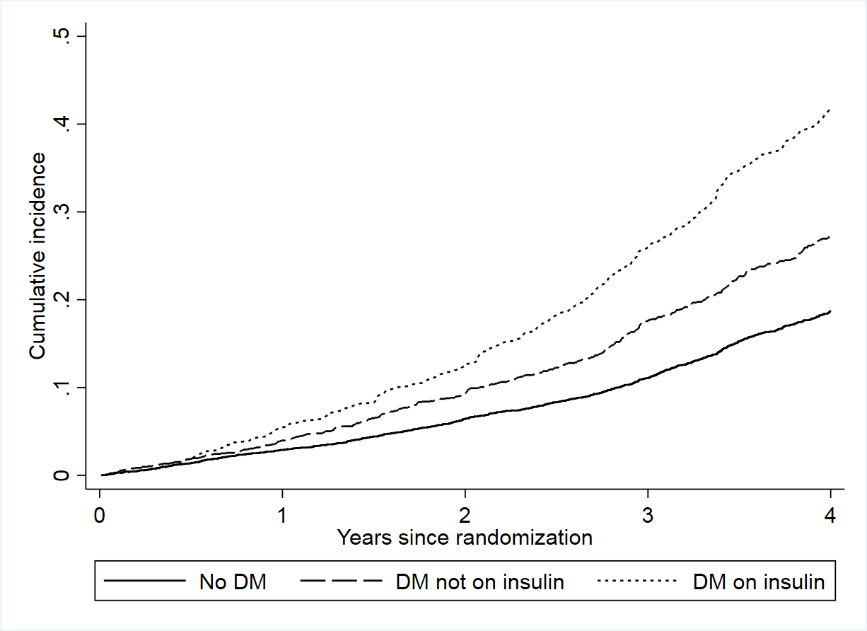

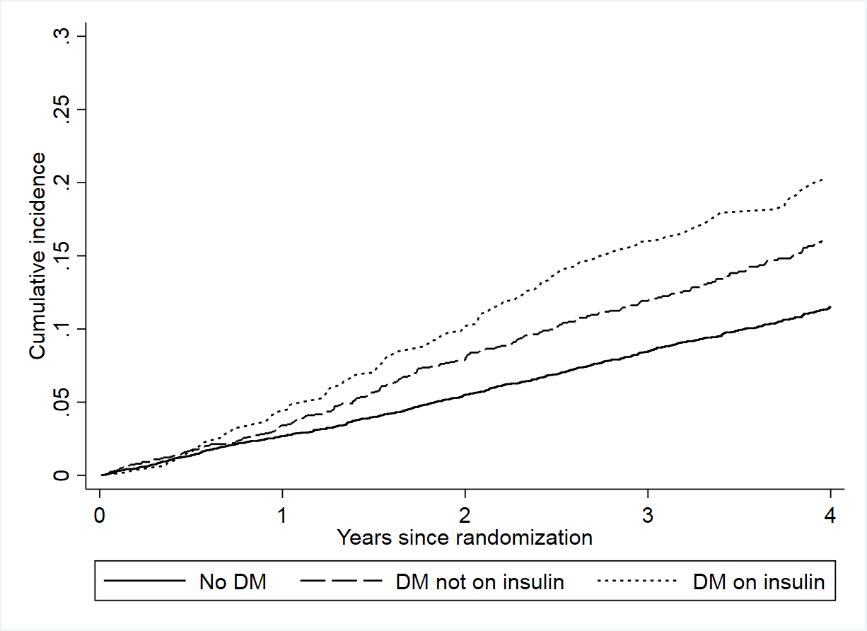


**B**

**A**


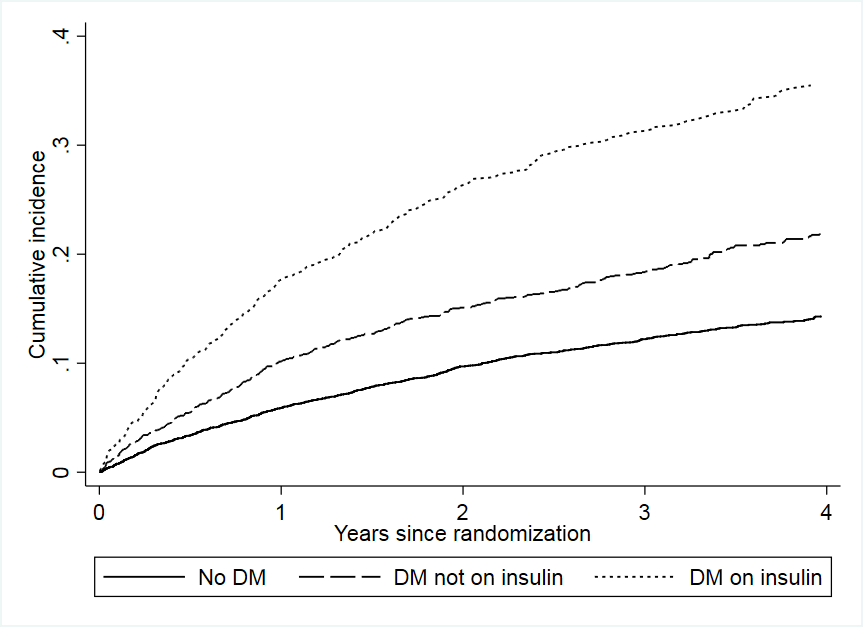

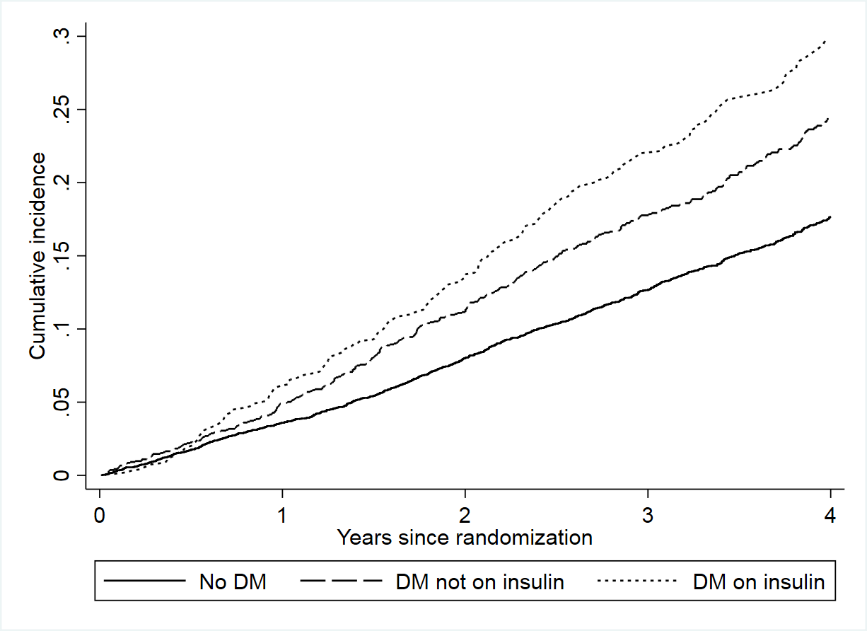


**C**

**D**


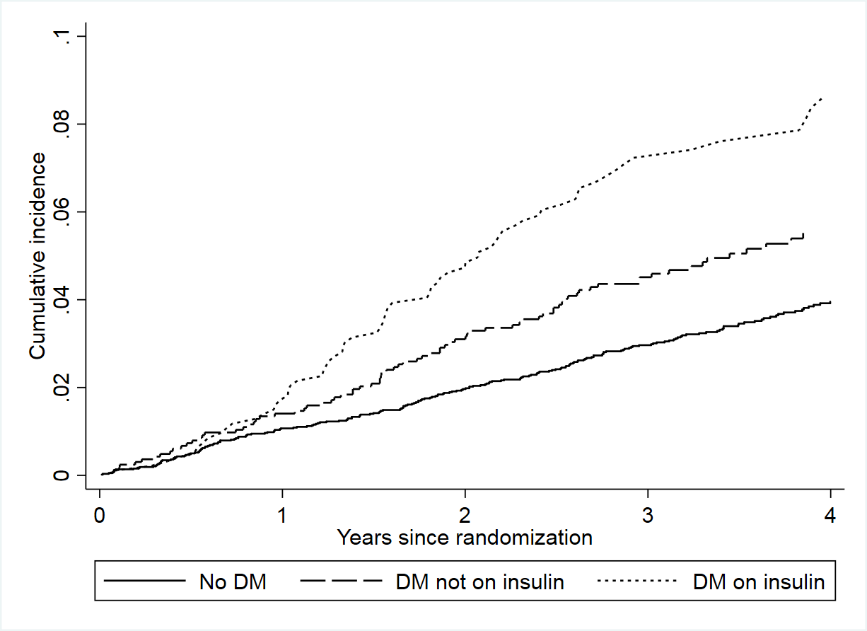

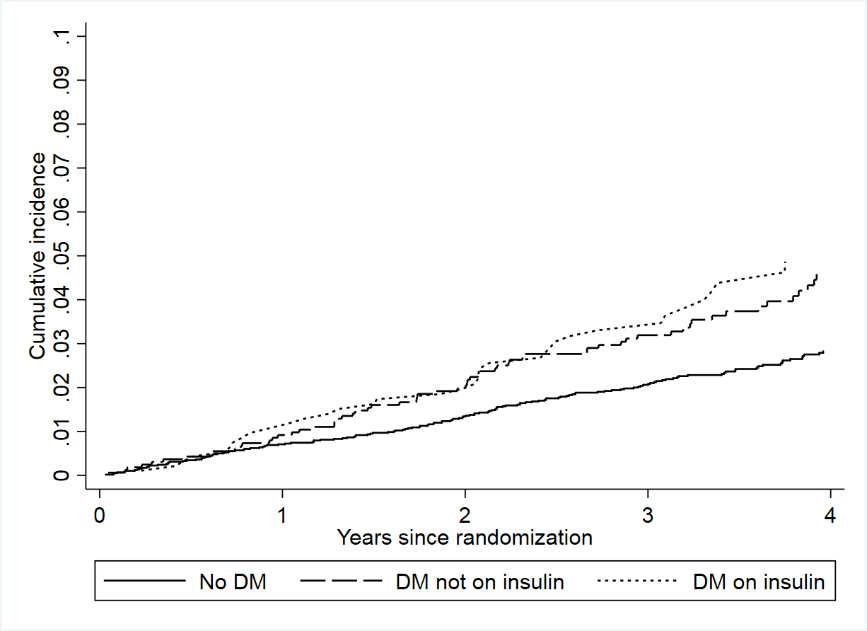


**F**

**E**

Cumulative incidences of the CV death or first hospitalization for HF (A), CV death (B), first hospitalization for HF (C), death from any cause (D), sudden death (E) and pump failure death (F). CV indicates cardiovascular; and HF, heart failure.
